# Simple fabrication of an electrospun polystyrene microfiber filter that meets N95 filtering facepiece respirator filtration and breathability standards

**DOI:** 10.1002/app.53406

**Published:** 2022-11-21

**Authors:** Madeline G. Jensen, Patrick T. O'Shaughnessy, Marlee Shaffer, Sooyoun Yu, Yun Young Choi, Megan Christiansen, Charles O. Stanier, Michael Hartley, Joey Huddle, Jed Johnson, Kyle Bibby, Nosang V. Myung, David M. Cwiertny

**Affiliations:** ^1^ Department of Civil and Environmental Engineering University of Iowa Iowa City Iowa USA; ^2^ Department of Occupational and Environmental Health University of Iowa Iowa City Iowa USA; ^3^ Department of Civil and Environmental Engineering and Earth Sciences University of Notre Dame Notre Dame Indiana USA; ^4^ Department of Chemical and Biomolecular Engineering University of Notre Dame Notre Dame Indiana USA; ^5^ Department of Chemical and Environmental Engineering University of California Riverside Riverside California USA; ^6^ Department of Chemical and Biochemical Engineering University of Iowa Iowa City Iowa USA; ^7^ Department of Hospital Administration University of Iowa Hospitals and Clinics Iowa City Iowa USA; ^8^ Nanofiber Solutions, LLC Dublin Ohio USA

**Keywords:** air filtration, antimicrobial, electrospinning, polystyrene

## Abstract

During the global spread of COVID‐19, high demand and limited availability of melt‐blown filtration material led to a manufacturing backlog of N95 Filtering Facepiece Respirators (FFRs). This shortfall prompted the search for alternative filter materials that could be quickly mass produced while meeting N95 FFR filtration and breathability performance standards. Here, an unsupported, nonwoven layer of uncharged polystyrene (PS) microfibers was produced via electrospinning that achieves N95 performance standards based on physical parameters (e.g., filter thickness) alone. PS microfibers 3–6 μm in diameter and deposited in an ~5 mm thick filter layer are favorable for use in FFRs, achieving high filtration efficiencies (≥97.5%) and low pressure drops (≤15 mm H_2_O). The PS microfiber filter demonstrates durability upon disinfection with hydroxyl radicals (•OH), maintaining high filtration efficiencies and low pressure drops over six rounds of disinfection. Additionally, the PS microfibers exhibit antibacterial activity (1‐log removal of *E. coli*) and can be modified readily through integration of silver nanoparticles (AgNPs) during electrospinning to enhance their activity (≥3‐log removal at 25 wt% AgNP integration). Because of their tunable performance, potential reusability with disinfection, and antimicrobial properties, these electrospun PS microfibers may represent a suitable, alternative filter material for use in N95 FFRs.

## Graphical abstract

The COVID‐19 pandemic led to a backlog of filtration material for N95 Filtering Facepiece Respirators (FFRs). An alternative face mask filter material is presented, consisting of an unsupported, nonwoven layer of uncharged polystyrene microfibers. This microfiber material meets NIOSH N95 FFR filtration and breathability requirements, demonstrates durability upon disinfection, and can be modified with silver nanoparticles for enhanced antibacterial activity.

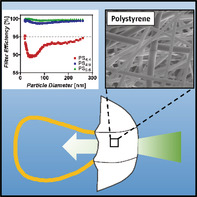

## 1 INTRODUCTION

The rapid emergence and spread of SARS‐CoV‐2, the causative virus for COVID‐19, led to a surge in the demand for personal protective equipment (PPE) for frontline healthcare workers. As an airborne respiratory virus, N95 Filtering Facepiece Respirators (FFRs) were highly sought after for protection from SARS‐CoV‐2 transmission via inhalation.^2^, ^3^ N95 FFRs are approved by the National Institute for Occupational Safety and Health (NIOSH) according to criteria stated in 42 CFR 84 of the Code of Federal Regulations.^4^ Briefly, they must be able to have a particle capture efficiency ≥95% when tested with a 75 nm NaCl aerosol at an inflow rate of 85 L/min. An N95 FFR should also induce a pressure drop at that inflow rate of ≤35 mm H_2_O. N95 FFRs are typically comprised of three layers of material, in which the second layer—a nonwoven layer of melt‐blown electret polypropylene—is the primary material responsible for filtration of aerosol.^5^ Unfortunately, a manufacturing backlog of this melt‐blown filtration material worsened N95 FFR shortages, causing global demand to far outpace the available supply.

The shortage of N95 FFRs has catalyzed interest in alternative methods to produce comparably performing filtration layers, thereby helping to alleviate future bottlenecks from nonwoven melt‐blown fibers. Electrospinning, a material fabrication process used to create non‐woven mats of nano‐ to micro‐fibers, has emerged as a popular approach because it is commercially viable at the industrial scale.^6^ Notably, electrospinning allows for tunable synthesis parameters that are foundational to filter performance including fiber diameter, the packing density of fibers in the filter layer, and the overall filter depth. Further, by relying on a simple sol–gel preparation process, the composition of the filter layer can be easily controlled. Thus, the type of filter material (e.g., hydrophobic versus hydrophilic), and the presence of performance‐enhancing additives (e.g., antimicrobial agents) and surface charge (e.g., through integration of cationic or anionic surfactants) can all be readily manipulated in response to application needs.

Prior to the COVID‐19 pandemic, many researchers explored the use of electrospinning to produce nonwoven layers for air filtration applications, including PPE development.^7^, ^8^, ^9^, ^10^, ^11^, ^12^, ^13^, ^14^, ^15^, ^16^, ^17^, ^18^, ^19^, ^20^, ^21^, ^22^ In response to COVID‐19, there has been renewed and increasing interest in electrospun materials both from the research community and private industry, including several commercially available masks and filters produced via electrospinning.^23^, ^24^ Most of these commercial products consist of a thin (~100 micron) layer of electrospun nanofibers deposited onto a more robust substrate. Many in the research community took a similar approach, electrospinning a thin polymer (e.g., polyvinylidene fluoride, polyvinyl butyral, polyacrylonitrile) nanofilter onto a support substrate (e.g., polypropylene spunbond) (Table S1).^7^, ^8^, ^15^, ^22^, ^25^, ^26^, ^27^, ^28^ Although this type of nanofiber‐supported platform has been reported to achieve particle capture efficiencies comparable to N95 FFRs, it requires the availability of an underlying support material because the thin, nanofiber layer is not sufficiently strong to be used by itself in such filtration applications.^9^ Accordingly, such supported nanofiber structures may remain vulnerable to supply chain limitations in times of public health crises if support substrates are not available in wide supply.^29^, ^30^, ^31^, ^32^ Alternatives to this supported nanofiber layer approach have therefore also been explored, such as nanofiber materials with more complex structures (e.g., multilayered, patterned structures, nanofiber‐network structures, hollowed fibers, fibers with nanoprotusions) (see material details in Table S1).^8^, ^9^, ^10^, ^13^, ^20^, ^22^, ^26^, ^33^, ^34^, ^35^, ^36^, ^37^, ^38^, ^39^, ^40^ While Lu et al.^13^ and Zhu et al.^22^ argue that these complex nanofiber materials are most effective at achieving high filtration efficiencies with low resistance, we note they often involve more complex synthesis (e.g., co‐electrospinning two or more polymers into a single layer) or additional processing of the fiber after electrospinning (e.g., imparting charge to the fiber) to enhance performance.^8^, ^10^, ^13^, ^20^, ^22^, ^26^, ^33^, ^34^, ^35^, ^36^, ^38^, ^39^, ^40^ Such complex processes take time and require additional raw materials, which may be unfavorable during public health emergencies.

To the best of our knowledge, a single‐layered, unsupported, electrospun filter made from larger (micro) fibers has yet to be developed, in which achieving N95 filtration performance relies only on physical parameters (e.g., filter thickness, fiber size, fiber packing density) that can be tuned during electrospinning (i.e., without additional post‐processing). Here, to address this gap, we have produced an unsupported filtration layer consisting of polystyrene (PS) microfibers with properties that were guided by modeled performance of N95 FFRs. We investigate microfiber filter performance changes in response to filter layer thickness, and ultimately identify a simple fabrication route to an unsupported filter layer that can achieve particle removal at or greater than N95 FFRs while also maintaining breathability that meets standards set by NIOSH.

Additionally, we show that this filter material is reusable after repeated disinfection with ionized hydrogen peroxide (iHP). Confronted with N95 FFR shortages, end users developed various approaches for disinfection that enabled their reuse.^41^ For example, the University of Iowa Hospitals and Clinics employed an advanced oxidation process that uses aerosolized hydroxyl radical (OH^•^) for N95 FFR decontamination and reuse. Research performed in response to COVID‐19 showed that this SteraMist™ process effectively inactivates SARS‐CoV‐2 as well as a variety of other pathogens (up to 6‐log inactivation in as little as 3 s), and that repeated disinfection (up to five use cycles) did not impact particle removal efficiency of N95 FFRs.^42^, ^43^, ^44^ Given the increasing use of such disinfection and reuse strategies for PPE in hospitals and other healthcare settings, we investigate if the filtration efficiency of electrospun PS microfibers changes over and beyond five disinfection cycles with iHP.

As an alternative to using periodic treatment with chemical disinfectants, another strategy to prolong the use of PPE while minimizing the exposure risk to pathogens accumulated on or in the filter media is to integrate antimicrobial agents into the PPE itself. Indeed, the research community has long sought to increase the functionality and extend the use of filter materials by introducing antimicrobial properties through the introduction of antimicrobial agents (e.g., quaternary ammonium salts [QASs],^45^ N‐halamines,^36^, ^46^ guanidine‐based polymers,^47^ antibiotics,^47^ metal oxides,^22^, ^36^, ^48^, ^49^ and metals^22^, ^36^, ^48^, ^50^, ^51^; see material details in Table S1). Among these agents, silver nanoparticles (AgNPs) have been used frequently in electrospun filter materials intended for use in N95 FFRs (see material details in Table S1).^8^, ^15^, ^20^, ^22^, ^36^, ^48^, ^52^, ^53^, ^54^, ^55^, ^56^, ^57^ However, the combination of electrospun PS microfibers with AgNPs has not yet been explored, motivating work herein to determine how integrated AgNPs influence the PS filter performance and assess antimicrobial activity of the AgNP‐modified microfiber filter toward *Escherichia coli (E. coli)*. Thus as a final objective to this work, we demonstrate that the PS microfiber filter layer developed herein can be easily tailored through its fabrication process to impart antimicrobial features (via the integration of AgNPs) while maintaining high filtration performance, thereby potentially avoiding the need for repeated treatment with chemical disinfectants.

## 2 MATERIALS AND METHODS

### 2.1 Reagents

Polystyrene (PS; avg. MW ~280,000 by GPC) and N,N‐Dimethylformamide (DMF; ACS reagent, ≥99.8%) were purchased from Sigma‐Aldrich, and silver nanoparticles (AgNP; avg. diameter of 80 nm) were purchased from Nanostructured & Amorphous Materials, Inc. All materials were used as received. For filter efficiency testing, a 2% NaCl solution was prepared by dissolving 2 g of NaCl (reagent grade, Sigma‐Aldrich) into 100 ml of distilled water. For antimicrobial testing, *E. coli* (ATCC 15597) was used, and the protocol required Phosphate‐Buffered Saline, 1X Without Calcium and Magnesium (VWR), Criterion m TEC Agar, and BD Difco™ Dehydrated Culture Media: LB Broth (Thermo‐Fisher Scientific).

### 2.2 Microfiber filter fabrication

Filter materials were fabricated using a previously described custom‐built electrospinning system (Figure S1).^58^ All filter materials were synthesized from a precursor solution of 30 wt% (with respect to total solution mass) polystyrene (PS) in N,N‐dimethylformamide (DMF). PS was selected as the base polymer for our materials for its hydrophobicity, which should help maintain material stability upon exposure to liquid or in high humidity environments caused by respiration.^59^, ^60^, ^61^ AgNPs were chosen as the means of incorporating biocidal properties into the PS filter given their easy integration into the precursor solution, low cost, and large surface area‐to‐volume ratio (which enhances silver's antimicrobial activity).^62^ For Ag‐amended materials, AgNPs were integrated into the PS precursor solution at 2 and 10 wt% loadings (also with respect to total solution mass) prior to electrospinning, resulting in PS materials with 6 and 25 wt% Ag final loadings, respectively.

Fabrication parameters are summarized in Table S2. Briefly, PS was first dissolved in DMF and thermally mixed (Thermostat) at 70°C for 30 h to create a homogeneous sol–gel as an electrospinning precursor solution. For Ag‐loaded sol–gels, AgNPs were first dispersed into DMF via sonication for 5 h, then PS was added and the solution thermally mixed using the conditions above. After cooling to room temperature, the sol–gel was transferred into a 12 ml syringe. The syringe was then mounted onto a syringe pump, which dispensed the sol–gel at a controlled rate of 1.2 ml/h. The sol–gel was expelled through an 18G metal needle tip connected to a high‐voltage power supply; the needle‐tip voltage was maintained at 29 kV during synthesis. Under the applied electric field, the sol–gel dispersed from the needle tip as fibers, which were collected on a grounded, rotating metal drum covered with aluminum foil. All electrospinning was performed in a custom environmental chamber to control the electrospinning environment at the desired temperature and humidity range: 21–25°C and 35%–40% RH, respectively. This range of RH is relatively high compared with our past work with polymer nanofibers^59^, ^63^, ^64^ (Greenstein et al. 2019; Peter et al. 2017) and was necessary to fabricate larger microfibers that were expected to be desirable based on models of N95 performance. Additional details on the materials, electrospinning system, and synthesis parameters can be found in Data S1.

### 2.3 Characterization

Viscosity and electrical conductivity of electrospinning precursor solutions were measured at room temperature immediately before electrospinning to correlate the measured values most closely to resulting microfiber properties. Chemical composition of electrospun materials were confirmed by Attenuated Total Reflectance Fourier Transform Infrared (ATR‐FTIR) spectroscopy using a Thermo‐Scientific Nexus 470 Fourier transform infrared spectrometer and a Smart Endurance ATR accessory. Morphology of the fibers was characterized by Scanning Electron Microscopy (SEM, ThermoFisher Prisma E). Micro‐scale elemental composition analysis was performed using Energy Dispersive X‐ray Spectroscopy (EDS, Thermo‐Fischer) to determine the distribution of AgNPs within composited fibers. SEM images were imported into ImageJ software (U.S. National Institutes of Health) to measure the fiber diameter, which was calculated by averaging 30 unique measurements.

Physical properties of the microfiber filters (e.g., filter thickness [Figure S2], volume, density, solidity) were assessed using at least three replicate batches of each material type, and are reported as averages with standard deviations. Tensile strength testing of the PS microfiber material at each studied thickness was assessed using a standard load–displacement testing apparatus (Universal Test Machine, Mecmesin) according to ASTM D638‐02a.^65^ Complete details of characterization instrumentation and methods can be found in Data S1.

### 2.4 Filter efficiency and pressure drop testing

Particle capture efficiency testing of the developed filter materials, N95 controls, and other filterable materials for this study generally followed the procedure described in 42 CFR 84.181 and as described in a NIOSH test standard for certifying N95 FFRs.^66^ Accordingly, this testing procedure will hereafter be referred to as the NIOSH N95 efficiency test. Pressure drop testing generally followed the procedure described in 42 CFR 84.180. Full procedural details and a schematic of the testing apparatus (Figure S3) are provided in Data S1.

### 2.5 Material disinfection

Material samples were disinfected via the decontamination process used at the University of Iowa Hospital and Clinics (UIHC) for decontaminating N95 FFRs.^42^, ^44^ Material samples were disinfected using a SteraMist™ Surface Unit (TOMI Environmental Solutions, Inc.), which produces a fine mist of ionized hydrogen peroxide (iHP).^67^ This approach for decontamination is an EPA‐registered (EPA Lists G, H, L, and M), hospital grade disinfectant since 2015.^42^ Complete procedural details can be found in Data S1.

### 2.6 Antimicrobial testing

Preliminary testing of the developed filter materials involved an assay similar to a diffusion ring test, where small portions of the filter material were placed on a spread plate. Results showed no evidence for diffusion, as *E. coli* colony growth was observed up to the material (i.e., no inhibitory zone). Following the outcome of this preliminary testing, methods for the assay were changed to analyze direct bacterial (*E. coli*) contact with the material. A complete description of the methods is provided in Data S1.

### 2.7 Microfiber filter handling simulation

To assess material durability, microfiber filter samples were manipulated to simulate two levels of handling during use, which we classified as “moderate” and “aggressive.” “Moderate” handling involved folding and twisting, while “aggressive” handling involved crumpling and abrasive rubbing (see Figure S4A,B, which provide images of the PS microfiber filter undergoing these handling procedures). A more detailed description of these handling methods can be found in Data S1.

### 2.8 AgNP retention evaluation

The retention of AgNPs within the PS fibers after handling was evaluated qualitatively (i.e., presence vs. absence) via XRD (Rigaku MiniFlex600). More quantitatively, we also estimated the surface area coverage of AgNPs on the 25 wt% Ag/PS material before and after handling procedures. This was accomplished using SEM with concentric backscatter (CBS) imaging. We report the surface area coverage of AgNPs on the fiber surface as a percentage (i.e., AgNP% values, calculated from the area attributable to AgNPs normalized to the total area of the Ag‐modified PS fibers in the image). Percentages are reported based on area calculations for 50 to 100 fibers per sample. Further details on these analyses can be found in Data S1.

## 3 RESULTS AND DISCUSSION

### 3.1 Microfiber filter characterization

Fabrication of the PS microfiber material was initially guided by modeling of N95 FFR performance (see summary in Data S1) assuming a single, active filtration layer made of fibers. Lu et al.^13^ suggest the ideal electrospun filter for effective particle filtration with easy airflow would have a small (e.g., nm) fiber diameter and high porosity. However, our modeling results suggested that N95 FFR performance could be accomplished with a filter of fibers ~10 μm in diameter and a filter depth of 300 μm. Because the model also considered other properties of the N95 FFR filter layer—including surface charge and particle density—these diameter and thickness values were only used as a rough guide for our material fabrication.

**FIGURE 1:**
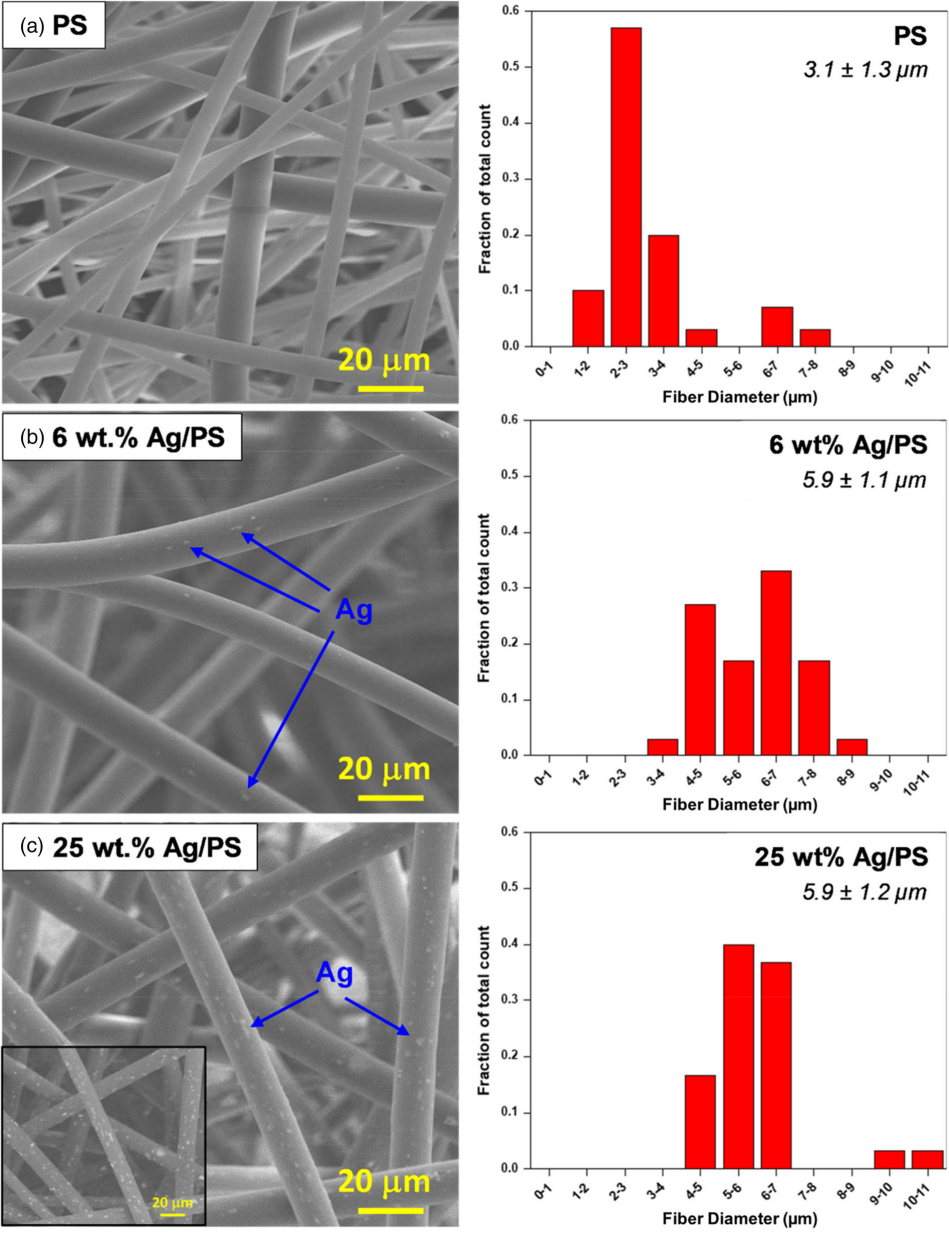
Representative images of (a) PS, (b) 6% Ag/PS, and (c) 25% Ag/PS materials. The inset found in (c) displays a BSE‐CBS image of the 25% Ag‐amended material. Corresponding histograms can be seen to the right of each image, showing the fiber diameter size distribution and average fiber diameter for each material. [Color figure can be viewed at wileyonlinelibrary.com]

Our fabrication method resulted in PS and Ag‐amended PS microfiber filters with a fluffy texture similar to quilt batting. ATR‐FTIR confirmed the composition of microfibers as polystyrene (Figure S5) based on previously published reference spectra.^1^ SEM images of electrospun PS microfibers revealed smooth fiber surfaces with an average diameter of 3.1 ± 1.3 μm (Figure 1a). This is comparable to the fiber diameters found in typical N95 filters (3–8 μm mean diameters).^68^, ^69^, ^70^ The integration of AgNPs increased fiber diameter by almost double (Figure 1b,c), an increase that was independent of the wt% of Ag integrated into the PS fibers. The electrical conductivity and viscosity of electrospinning sol gel solutions are known to influence the diameter of resulting fibers, thus we ascribe this increase to measured changes in these sol gel properties in response to Ag addition (see Table S3).^71^, ^72^ As shown in Figure 1, AgNPs were clearly observed (see blue arrows in Figure 1b,c) throughout the fibers, with some at or near the surface.

Micro‐scale elemental composition analysis and color SEM imaging were performed to qualitatively characterize the distribution of AgNPs along the fibers (Figure S6). Bulk quantitative analysis of the AgNPs was of limited value as the only other element for comparison was carbon, which is typically not accurately quantified via EDS due to its low atomic number. As expected, AgNPs were detected at higher density for materials with 25 wt% Ag relative to those with 6 wt% (compare Figure S6‐A and S6‐B).

Cross‐sectional images of nonwoven PS microfiber layers of varying thickness, produced from 3.5‐, 4‐, and 4.5‐ml sol–gels of 30 wt% PS in DMF, are shown in Figure S2. Table 1 provides the corresponding nonwoven PS layer thicknesses for each material. As expected, increasing the volume of sol–gel electrospun and collected on our rotating drum collector resulted in a corresponding increase in the thickness of the filter layer. Typically, filter materials were between 4.4 and 5.6 mm thick, increasing by roughly 0.5 mm in thickness per 0.5 ml increase in sol–gel volume. For simplicity, we hereafter refer to these filters based on their thicknesses as PS_4.4_, PS_4.9_, and PS_5.6_. These filters have significantly thicker layers than typically seen for N95 filters, which are usually 0.5–1.5 mm thick.^68^, ^69^

**TABLE 1:** Physical properties of PS materials produced from different sol–gel volumes and amended with different weight percentages of AgNPs

Material type	Sol–gel volume (ml)	Avg. mass (g)	Layer thickness (mm)	Avg. area (cm^2^)	Avg. volume (±σ, cm^3^)	Avg. filter density (±σ, g/cm^3^)	Avg. solidity
PS	3.5	0.99 ± 0.05 (*n* = 4)	4.4 ± 0.7 (*n* = 3)	417 ± 3 (*n* = 4)	180 ± 30	(5 ± 0.9) × 10^−3^	(5 ± 0.8) × 10^−3^
	4	1.14 ± 0.05 (*n* = 6)	4.9 ± 0.7 (*n* = 4)	415 ± 9 (*n* = 6)	200 ± 30	(6 ± 0.9) × 10^−3^	(5 ± 0.9) × 10^−3^
	4.5	1.28 ± 0.04 (*n* = 3)	5.6 ± 0.8 (*n* = 3)	381 ± 30 (*n* = 3)	210 ± 40	(6 ± 1) × 10^−3^	(6 ± 1) × 10^−3^
6 wt% Ag/PS	4	1.16 ± 0.10 (*n* = 4)	3.8 ± 0.6 (*n* = 3)	410 ± 1 (*n* = 4)	160 ± 20	(7 ± 1) × 10^−3^	(5 ± 0.8) × 10^−3^
25 wt% Ag/PS	4	1.5 ± 0.2 (*n* = 3)	4.1 ± 0.8 (*n* = 3)	400 ± 5 (*n* = 3)	160 ± 30	(9 ± 2) × 10^−3^	(3 ± 0.6) × 10^−3^

Table 1 also includes additional physical details (e.g., average mass, volume, and density) of the PS materials produced from each of the sol–gel volumes explored. As expected, mass of the PS filter material increased in correspondence to higher volumes of sol–gel electrospun. On average, filter mass increased by about 0.14 g per 0.5 ml increase in sol–gel volume. Because all sol–gels were electrospun and collected using the same rotating drum collector, the areas of the deposited PS materials remained relatively consistent across all sol–gel volumes. The PS layer thickness was therefore the determining factor in the PS material volume, leading to increased material volume with layer thickness. Filter density, and thus solidity (or “packing density”), of the deposited PS layers remained relatively consistent regardless of the volume of sol–gel electrospun (Figure S7), suggesting solidity remains constant no matter the material thickness. It should be noted that when compared with the solidity of typical N95 filters (0.05–1.0), the solidity of the PS microfiber material is smaller by at least one order of magnitude.^68^, ^69^ Since solidity is the inverse of porosity, this indicates that the porosity of the PS microfiber material is greater than typically found in N95 filters.

Material strength results of the PS microfiber filter are presented in Table S4 and compared to the meltblown polypropylene fibers used in a certified 3M N95 mask. At each thickness, the PS filter materials exhibit an ultimate tensile strength that either meets or exceeds that measured for the meltblown polypropylene fibers (Table S4). Based on measured values for elongation and modulus, the PS materials also tend to be more flexible than the 3M material. Durability of the PS, 6 wt% Ag/PS, and 25 wt% Ag/PS filters was also qualitatively assessed during and following moderate and aggressive handling procedures. All three filter materials successfully withstood several repetitions of our moderate handling procedure (i.e., folding and twisting), with only minor deterioration of the outer fiber layers observed (Figure S4C–E). While considerable damage of the outermost fiber layers was observed after the aggressive handling procedure (Figure S4C–E), the filter material largely remained intact. Overall, these results demonstrate that the developed PS microfiber material (both with and without Ag integration) is strong enough for its intended application as an alternative filter material to be integrated into N95 FFRs.

### 3.2 Material filtration and pressure drop performance

**FIGURE 2:**
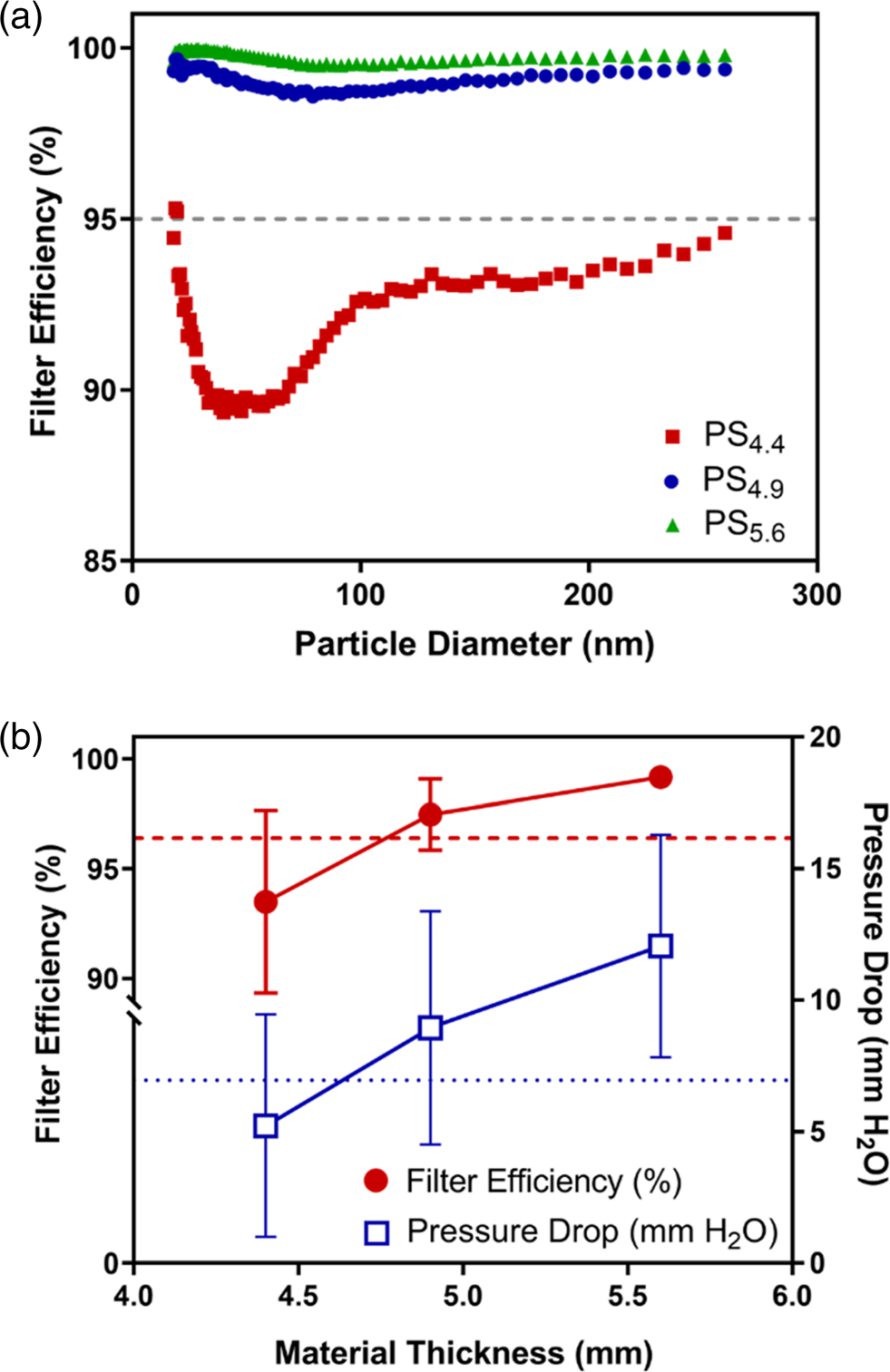
(a) Efficiency curves for PS microfiber material at 4.4 ± 0.7 mm, 4.9 ± 0.7 mm, and 5.6 ± 0.8 mm thicknesses (PS_4.4_, PS_4.9_, and PS_5.6_, respectively). One representative efficiency curve is shown per thickness to illustrate the filter efficiency data collected. (b) Average filter efficiency and pressure drop for PS_4.4_, PS_4.9_, and PS_5.6_ microfiber materials; the averages and corresponding standard deviations are derived from triplicate measurements for each layer thickness. The larger and smaller dotted lines represent the filter efficiency (96.4%) and pressure drop (6.96 mm H_2_O), respectively, measured for an N95 FFR. [Color figure can be viewed at wileyonlinelibrary.com]

Figure 2a shows representative efficiency curves based on capture of NaCl aerosol for PS_4.4_, PS_4.9_, and PS_5.6_ microfiber mats. These curves illustrate how well a material filters out specific particle sizes comprising the NaCl aerosol used for NIOSH N95 efficiency testing. The dip seen in each efficiency curve occurs in a region that encompasses the particle size that most penetrates the filter material (MPPS, or Most Penetrating Particle Size). The minimum efficiency and MPPS provide useful insight into the forces affecting particle deposition on these filter materials (e.g., diffusion on smaller particles and interception and impaction on larger particles; see, e.g., Figure S8). Additionally, these data were used to calculate the total filter efficiency of a material (see methods), which is the combined filter efficiency for all particle sizes of the NaCl aerosol and the parameter used in the NIOSH certification method for N95 FFRs.

Figure 2b presents averages for the total filter efficiency—as well as the corresponding pressure drop across the filter—of the PS_4.4_, PS_4.9_, and PS_5.6_ microfiber layers. These averages and corresponding standard deviations were derived from triplicate filter efficiency tests and pressure drop measurements for each PS layer thickness. It should be noted that each replicate came from separate batches of the same fabrication recipe, demonstrating that these materials can be reproducibly fabricated from day to day. In Figure 2b, our results reveal the expected relationship, in which increasing material thickness leads to improved filtration efficiency. For PS microfiber layers equal to or greater than 4.9 ± 0.7 mm in thickness, these electrospun PS materials achieve the minimum total filtration efficiency required by NIOSH for N95 FFRs, with efficiencies of 97.5 ± 1.6% and 99.2 ± 0.5% for PS_4.9_ and PS_5.6_, respectively. Pressure drop, which represents the breathing resistance of the material, likewise increases with material thickness. For instance, a ~ 30% increase in PS layer thickness (PS_4.4_ to PS_5.6_) causes the pressure drop to more than double from ~5 to ~10 mm H_2_O. Thus, as is often observed with different materials, increases in filtration efficiency (and more aerosol removal) come at the expense of breathability. Indeed, the maximum pressure drop allowed by NIOSH for N95 FFRs is 35 mm H_2_O (353 Pa) for inhalation and 25 mm H_2_O (245 Pa) for exhalation.^4^, ^73^ However, the nonwoven PS layers created as part of this research achieved greater than 95% filtration efficiency while remaining well below these limits.

We also performed filter efficiency and pressure drop testing on the PS_4.9_ material after conducting our moderate and aggressive handling procedures (Figure S9). In fact, PS_4.9_ filtration efficiency increased after both moderate and aggressive handling, likely due to material compaction as a result of bending, folding, and crumpling, although the pressure drop across the handled filter materials remained comparable to that measured for pristine (without handling) filters.

The filtration efficiency and pressure drop performance exhibited by the PS microfiber materials are comparable, if not better, to the performances of the supported, polymer‐based electrospun materials previously developed with the intent of being alternative materials for use in N95 FFRs. The two polypropylene spunbond‐supported cellulose acetate nanofilters (~300 nm fiber diameters) developed by Akduman^7^ that met NIOSH requirements for N95 masks achieved similar filtration efficiencies to our PS microfiber filter (~95%); however, both exhibited pressure drops around 30 mm H_2_O, which is significantly higher than those of the PS microfiber filters. The hybrid PS/PVDF microfiber material (14/2 mass ratio; 3–4 μm fiber diameter) created by Li et al.^35^ generated a pressure drop (~7 mm H_2_O) similar to those of the PS microfiber filters. However, prior to modification this material produced only an 83.7% filtration efficiency, which is about 10% lower in efficiency compared to PS_4.4_. Only after charge injection into the fibers did filtration efficiency improve to 99.7%, which is on par with the performance of PS_5.6_.^35^ It should also be noted that charge on fibers can decrease over time, particularly during decontamination, leading to decreases in mask filtration efficiency.^74^ The modified PS microfiber materials developed by Jung et al.^34^ are most similar to those produced herein, with a 2–3 μm fiber diameter but an average layer thickness of 0.13 mm. Although these materials had pressure drops (6.4–6.8 mm H_2_O) similar to our PS microfiber filters, they achieved far lower filtration efficiencies (31.4%–86.5%) that do not meet NIOSH requirements for N95 masks.^34^ According to Jung et al.,^34^ the porosities of their fibers were all around 90%, suggesting a solidity around 0.1 that is greater than that calculated for our PS microfiber filters. Because higher solidity should result in higher filtration efficiency,^75^ we presume the lower filtration efficiency of the PS materials in Jung et al.^34^ must be due to their significantly smaller layer thickness.

We also compared the filter efficiency and pressure drop results for the microfiber PS filters to the performance of a certified N95 FFR (Figure 2b) and several commercially available electrospun filters produced by commercial vendors in response to COVID‐19 (Table 2). PS_4.9_ and PS_5.6_ exhibit equal or higher efficiencies relative to the N95 FFR (at 96.4%), albeit at slightly higher pressure drops and, thus, somewhat lower breathability than the N95 FFR (6.96 mm H_2_O). The results for the commercially available electrospun filter materials can be seen in Table 2 (please note we have elected not to disclose the name of the product vendor(s)). Nearly all tested commercially available electrospun materials exhibited filtration efficiencies well below the performance required for N95 FFRs by NIOSH. Only Material 4, a polyvinylidene fluoride (PVDF) copolymer on polypropylene blue spunbond, exceeded the minimum efficiency requirement for N95 FFRs. However, this was achieved at an extremely high pressure drop (> 67 mm H_2_O) that far exceeds the NIOSH breathability standard, making it a poor choice for a mask or respirator material. Additionally, a member of the study team (P.T.O.) used the apparatus described here to test other commercially available filter media (e.g., melt‐blown and polypropylene spunbond‐supported materials), the details and results of which can be found in O'Shaughnessy et al.^76^ There, only 2 of the 14 filter media tested achieved filtration efficiencies ≥95%, with the rest holding efficiencies around 70%–90%. The two most efficient filter media exhibited pressure drops at 7.5 and 10.6 mm H_2_O, showing comparable performance to those of our PS microfiber filters.^76^ Thus, based on the performance standards for N95 FFRs, and relative to commercially available materials (electrospun and other), our PS microfiber filters represent a reasonable alternative for N95 FFR development.

**TABLE 2:** Total filtration efficiency and pressure drop results for industrially‐made electrospun filter materials

Industrial material	Filter efficiency (%)	Pressure drop (mm H_2_O)
Material 1 (*n* = 1)	82.0	3.76
Material 2 (*n* = 1)	65.0	3.77
Material 3 (*n* = 1)	71.3	3.19
Material 4 (*n* = 1)	100.0	>67
Material 5 (*n* = 1)	84.1	5.54

### 3.3 Disinfection and reuse of PS microfiber filters

**FIGURE 3:**
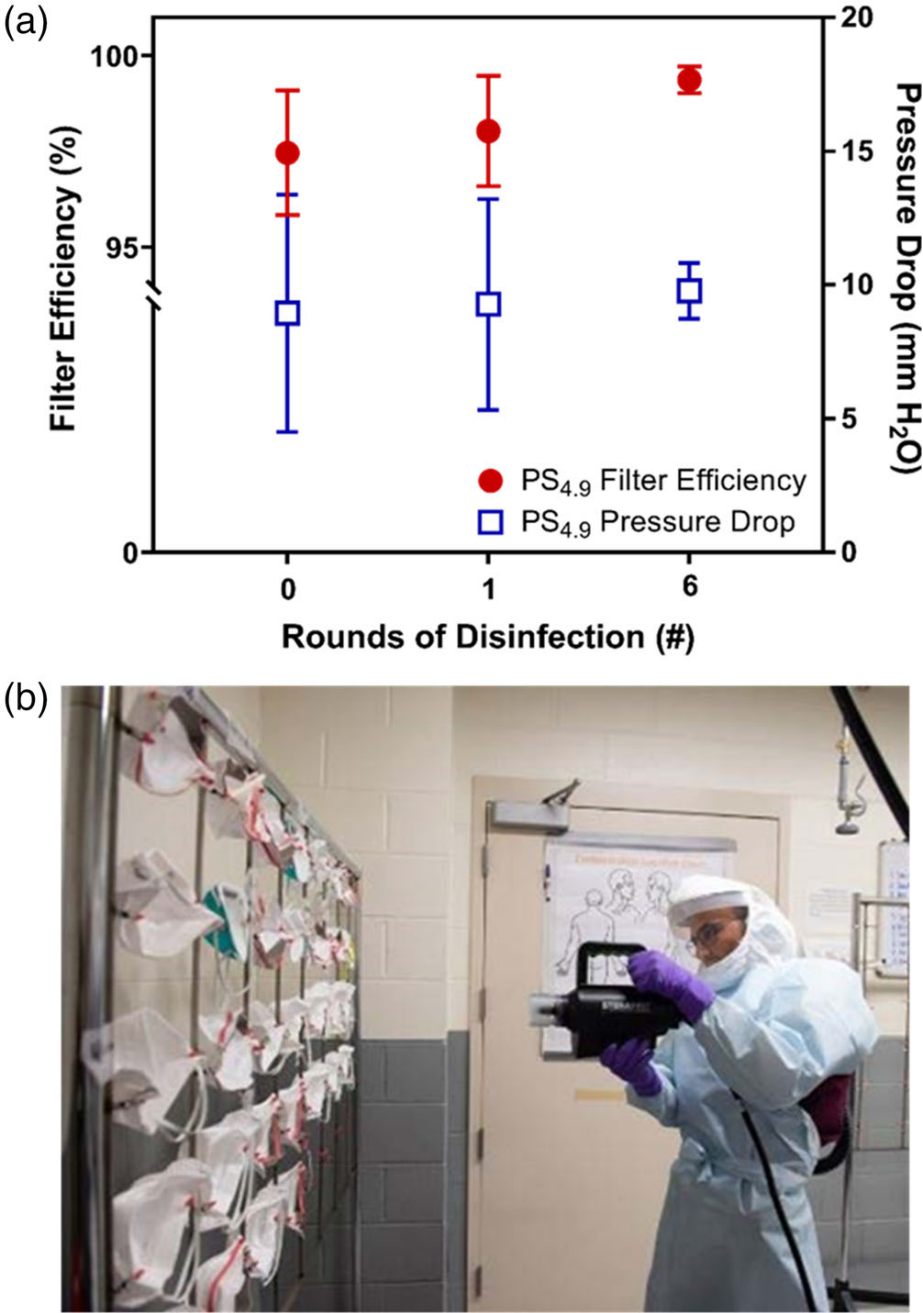
(a) Filter efficiency and pressure drop of PS_4.9_ microfiber materials after zero (*n* = 3), one round (*n* = 3), and six rounds (*n* = 3) of disinfection with iHP. (b) Snapshot of the mask disinfection process used at UIHC in which used N95 masks were sprayed with aerosolized hydroxyl radical (iHP) from a SteraMist spray device. [Color figure can be viewed at wileyonlinelibrary.com]

Based on the desire to achieve adequate filtration performance while minimizing pressure drop across the filter material, all subsequent testing was conducted with the optimal PS_4.9_ material. Figure 3 shows the filter efficiency performance of PS_4.9_ materials after undergoing one and six rounds of disinfection with iHP. For comparison, work by others^42^ and previous testing in this laboratory (unreported) applied to the same decontamination system demonstrated that N95 FFR particle capture efficiency was unaffected by up to 10 decontamination cycles on the same FFR.

From the results shown in Figure 3, the PS_4.9_ material shows no loss in performance after six rounds of disinfection, with pressure drops (~10 mm H_2_O) remaining well below those allowable by NIOSH and filter efficiencies above the minimum 95% required for N95 FFRs in 42 CFR 84.180.^4^, ^73^ These results are consistent with established behavior regarding the stability of PS toward chemical oxidants and other disinfectants. PS is known to have good resistance against hydrogen peroxide and is stable during sterilization via ethylene oxide and gamma radiation.^60^, ^61^ Indeed, under gamma radiation the high aromatic content of PS absorbs the radiation and eliminates the creation of reactive free radicals.^60^, ^61^

It should also be noted that the decontamination process using iHP involved multiple wetting and drying cycles, with visible accumulation of liquid on the filter surface when wet. Thus, based on the reusability of these materials after disinfection with iHP, we anticipate that use of other, more common spray disinfectants would also be sufficient for disinfection of the filter material so long as the material is adequately wetted. Additionally, while we did not test material integrity during simulated mask use at high relative humidity of human breath, our disinfection results imply the PS_4.9_ material will be sufficiently robust to maintain integrity and performance under such conditions.

### 3.4 Filtration efficiency and antimicrobial properties of Ag‐containing PS filters

**FIGURE 4:**
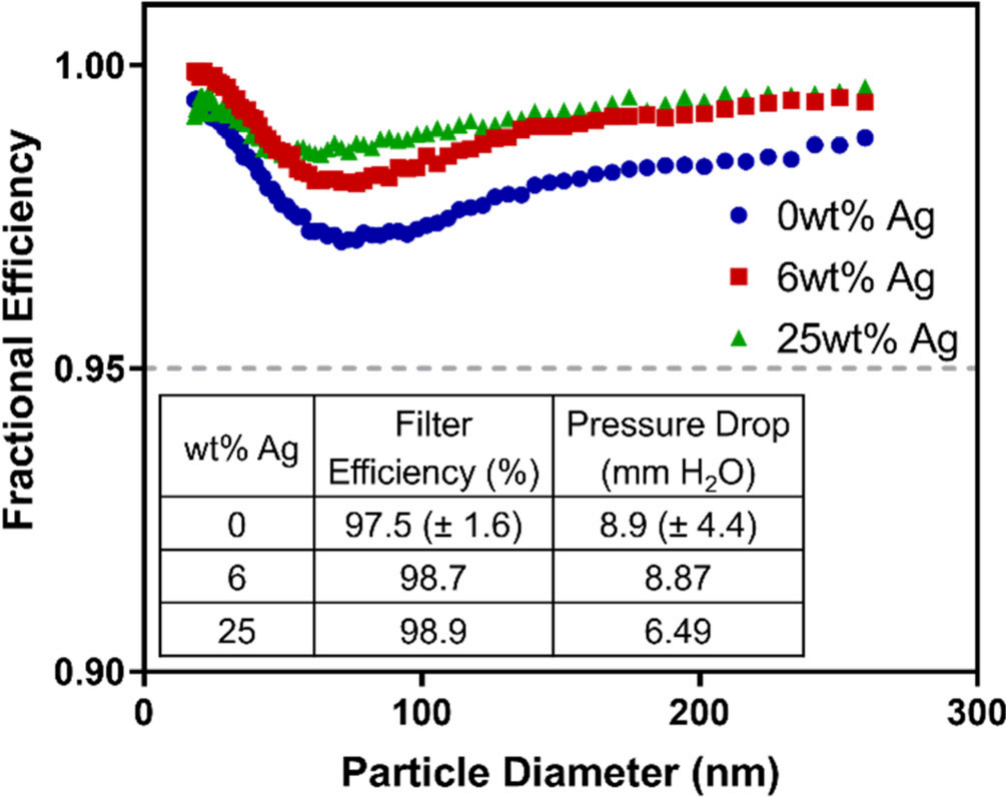
Efficiency curve comparison of PS_4.9_ filter material with Ag nanoparticle integration. AgNPs were integrated into the PS_4.9_ recipe at 6 and 25 wt%. Ag‐amended materials were compared with the standard PS_4.9_ material recipe. Here, only one representative efficiency curve for PS_4.9_ is shown [Color figure can be viewed at wileyonlinelibrary.com]

As an alternative disinfection route to chemical decontamination processes, we explored the integration of AgNPs into the PS microfiber material to promote the inactivation of pathogens captured on or within the filter material. Figure 4 shows representative filter efficiency curves for the standard PS_4.9_ microfiber material relative to two forms of PS amended with AgNP at different wt% (6 and 25 wt%). Data show that the integration of Ag does not negatively impact performance in terms of filtration efficiency and pressure drop. As summarized in the table inset of Figure 4, the efficiencies and pressure drops of the Ag‐amended materials are comparable to that of the standard PS_4.9_ microfiber material, if not better. This is notable because the inclusion of AgNPs increased the diameter of the PS microfibers from ~3 to 6 μm (see Figure 1), while the average layer thicknesses of the Ag‐amended materials are smaller than PS_4.9_ by roughly 1 mm (Table 1). Moreover, the solidities of the Ag‐amended materials are lower than that of PS_4.9_ and decrease with increasing Ag wt% (Table 1). Collectively, the smaller layer thicknesses, lower solidities, and increased fiber diameters of the Ag‐amended materials would suggest that a decrease in filtration efficiency and pressure drop should be anticipated.^75^, ^77^ Although we do observe a decrease in pressure drop, especially for the 25 wt% Ag material, we do not see any corresponding decrease in filtration efficiency. This observation may be explained by the change in fiber morphology from the integration of the AgNPs (see Figure S6)—adding “roughness” to the fiber morphology has been shown to improve filtration efficiency.^49^, ^77^ Further investigation into this matter is needed.

We also conducted our moderate and aggressive handling procedures on both Ag‐amended materials followed by filter efficiency and pressure drop testing (Figure S9). For both Ag‐amended materials, filtration efficiency remained well above 95% after both moderate and aggressive handling. Filtration efficiency generally increased after handling, with the exception of 25 wt% Ag/PS showing a slight decrease in efficiency after aggressive handling. Similar to PS_4.9_, this is likely due to compaction from folding and crumpling. Pressure drops remained comparable or lower (in the case of the 25 wt% Ag/PS filter) to that of pristine materials.

AgNP retention within the Ag‐amended filters was evaluated by comparing the surface area coverage of AgNPs before and after handling for a 25 wt% Ag/PS filter. Surface area coverage of AgNPs (AgNP% values) determined from SEM analysis are presented in Table S5. Our analysis indicates tthere is no statistically significant loss of AgNPs from the fibers after both levels of handling, as the AgNP% values for all three handled (pristine, moderate, aggressive) filters are within one standard deviation of each other (8.6 ± 0.9, 6.2 ± 1.9, and 9.1 ± 1.2 respectively). Likewise, the XRD patterns for all three handled filters (Figure S10) exhibited clear diffraction lines for metallic silver, also supporting retention of AgNPs in the material after handling.

**FIGURE 5:**
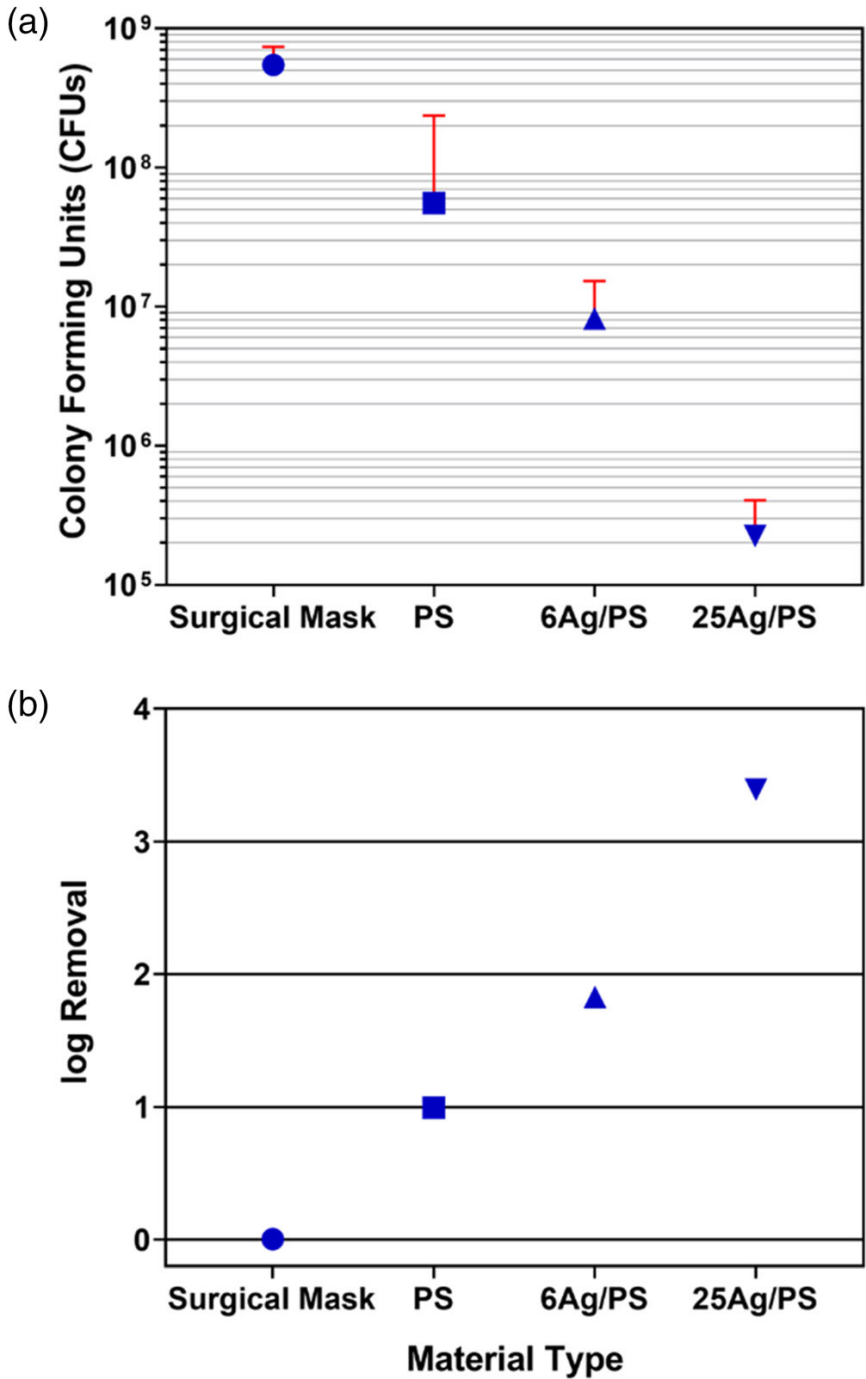
(a) Colony forming units (CFUs) measured on each material type. Data represent the mean CFU of the triplicate readings with an error bar showing the standard deviation. (b) Calculated log removal of *E. coli* on each material type. [Color figure can be viewed at wileyonlinelibrary.com]

Figure 5a shows the average colony growth and upper bound of the standard deviation for the PS_4.9_ and Ag‐amended filter materials tested for antimicrobial activity using *E. coli*. The error bars shown include the difference in the triplicate readings and incorporate a +/− 5 CFU for human error in counting. A standard, non‐medical grade, 3‐ply polypropylene disposable surgical mask^78^ was also tested as a control comparison; all three layers were tested. The surgical mask supported the highest amount of bacterial growth, with an average of (5.5 ± 1.9) × 10^8^ CFU. Each of the developed PS microfiber materials had less growth than the surgical mask, with bacterial growth on PS_4.9_, 6% Ag/PS, and 25% Ag/PS of (5.60 ± 0.18) × 10^7^ CFU, (8 ± 7) × 10^6^ CFU, and (2.2 ± 1.8) × 10^5^ CFU, respectively. Figure 5b illustrates the log removal of *E. coli* grown on the filter substrates relative to the *E. coli* colonies in the initial broth. The initial OD 600 reading was 0.689, and the plate counts showed colony growth of (5.5 ± 1.0) × 10^8^ CFU. The data indicate that the PS microfiber filters have substantial antibacterial properties when compared with a standard surgical mask, and that the extent of antimicrobial activity can be increased by the amount of Ag added to the PS microfibers. Log removal of *E. coli* jumped from 1‐log removal by the unmodified PS_4.9_ material to more than 3‐log removal by the 25% Ag/PS material.

The positive trend observed for *E. coli* inactivation with increased AgNP loading is in agreement with the findings by Palomba et al.,^79^ who saw increased inhibition of *E. coli* growth with higher concentrations of AgNP embedded in PS films. The proposed mechanism responsible for the antimicrobial properties of AgNPs is their continual release of Ag^+^ ions, which adhere to and ultimately break through cell walls and cytoplasmic membranes; after this initial breakthrough, the Ag^+^ ions suppress cell respiration and growth and can eventually cause cell lysis.^22^, ^62^, ^80^ Thus, the antimicrobial mechanism of AgNPs depends on bacterial proximity and the capability of released Ag^+^ ions to reach cell walls. Indeed, Palomba et al.^79^ determined that the antibacterial activity of AgNPs is increased when there is a higher amount of AgNP at the surface relative to the bulk of their material. There is an appreciable amount of AgNPs found at or near the surface of the microfibers in our Ag‐amended materials—especially for the 25 wt% Ag/PS material (see Figures 1 and S6)—which likely contributes to their antimicrobial activity.

The considerable amount of antimicrobial activity displayed by the unmodified PS microfiber filter is also noteworthy. Other studies have demonstrated the antimicrobial activity of PS particulates and nanoparticles against microbiota in soil and water.^81^, ^82^, ^83^ According to the studies by Yamamoto et al.,^82^, ^83^ the toxicity of PS against the microorganism *Micrococcus luteus* is dependent on its molecular weight (≤1000 g/mol) and morphology (≤150 nm in size), as these parameters allow for penetration of the cell membrane. Our PS microfiber materials do not fit these parameters: we used 280,000 MW polystyrene, and our fibers are much larger than 150 nm in any dimension. However, Abrigo, Kingshott, and McArthur^84^ determined how the morphology of electrospun PS fibers specifically can affect bacterial growth. In particular, electrospun PS fibers with diameters much smaller or larger than a bacteria's cell size will limit cell growth to the fiber surface, preventing colony formation and its accompanying protection and leading to cell death; these effective fiber diameters are dependent on the size and shape of the bacteria in question.^84^ We presume the antimicrobial activity demonstrated by the unmodified PS microfiber filter against *E. coli* is due to something similar, but further investigation is required.

## 4 CONCLUSIONS

In this work, we produced via electrospinning an alternative filter material to be integrated into N95 FFRs consisting of polystyrene microfibers, which hold several benefits over other alternative electrospun filters. Fabricated as a thick, single‐layered filter, our PS microfiber material can be used in N95 FFRs or in other masks as a standalone filter insert, removing the need for a supporting layer. This may be advantageous during supply‐chain shortages of common support materials (i.e., spunbound polypropylene) as was seen during the COVID‐19 pandemic. Notably, we produced these filter materials with minimal reagents (only PS and DMF), which should also help these filters be less prone to supply chain limitations during public health emergencies. By adjusting only physical properties of the material (e.g., thickness)—which can be easily tuned during synthesis—these filters are able to achieve efficiencies and breathability that meet or exceed the NIOSH requirements for N95 FFRs (the “gold standard” for air filtration during public health crises). Indeed, N95 FFRs require a charged (electret) material (meltblown polypropylene) as the primary filter layer; in contrast, our PS microfiber filters achieve comparable or better performance in a single, uncharged filter layer.

We further demonstrate the robustness and reusability of our PS microfiber filter material, as it maintained its filtration efficiency and pressure drop performance through six disinfection cycles with iHP. Thus, the material is suitable for use at healthcare facilities using disinfection techniques to sterilize and reuse PPE. We also found the materials to withstand aggressive handling without compromising its performance. Accordingly, these PS‐based materials appear to be sufficiently robust for everyday use. Finally, as an alternative to treatment with chemical disinfectants, we show that we can impart antimicrobial properties to our PS microfiber filter material with the integration of AgNPs via electrospinning, which may also increase the functionality and reusability of the filter material without sacrificing its breathability and filtration performance.

While these results are promising, more work is certainly needed to integrate these materials into wearable PPE and test durability and performance during use by healthcare workers. This will require translating the fabrication method developed herein to larger electrospinning rigs that can produce industrial scale nonwoven, microfiber layers. These filter layers will then need to be inserted into a mask or FFR as the primary filtration layer with active filtration area on par with a conventional N95 FFR. For Ag‐modified materials, their universal antimicrobial properties need to be further assessed, with future antimicrobial evaluations including additional targets (e.g., viruses and Gram‐positive bacteria). Finally, while we conducted a preliminary assessment of Ag stability within the PS fibers, additional studies will be needed to ensure there are no unintended health effects associated with the use of Ag particles in the masks. Nevertheless, we contend that the PS microfiber material developed in this work holds advantages over other proposed alternatives to melt‐blown fiber filters by not requiring an underlying support material, and having the ability to extend filter lifetime—without sacrificing filtration efficiency and breathability—via simple integration of antimicrobial activity or through repeated disinfection.

## AUTHOR CONTRIBUTIONS

**Madeline G. Jensen:** Data curation (lead); formal analysis (lead); investigation (equal); methodology (supporting); writing – original draft (lead); writing – review and editing (lead). **Patrick O'Shaughnessy:** Conceptualization (equal); data curation (supporting); formal analysis (supporting); funding acquisition (supporting); investigation (supporting); methodology (equal); resources (supporting); supervision (supporting); writing – original draft (supporting); writing – review and editing (supporting). **Marlee Shaffer:** Data curation (supporting); formal analysis (supporting); investigation (supporting); methodology (supporting); resources (supporting); writing – original draft (supporting); writing – review and editing (supporting). **Sooyoun Yu:** Data curation (supporting); formal analysis (supporting); investigation (supporting); methodology (supporting); writing – original draft (supporting); writing – review and editing (supporting). **Yun Young Choi:** Data curation (supporting); formal analysis (supporting); investigation (supporting); writing – original draft (supporting); writing – review and editing (supporting). **Megan Christiansen:** Data curation (supporting); formal analysis (supporting); investigation (supporting); methodology (supporting); writing – original draft (supporting). **Charles O. Stanier:** Formal analysis (supporting); investigation (supporting); methodology (supporting); resources (supporting); writing – original draft (supporting); writing – review and editing (supporting). **Michael Hartley:** Formal analysis (supporting); investigation (supporting); methodology (supporting); resources (supporting); supervision (supporting); writing – original draft (supporting). **Joey Huddle:** Data curation (supporting); formal analysis (supporting); investigation (supporting); methodology (supporting). **Jed Johnson:** Investigation (supporting); methodology (supporting); resources (supporting); writing – original draft (supporting). **Kyle Bibby:** Conceptualization (supporting); data curation (supporting); formal analysis (supporting); funding acquisition (equal); investigation (supporting); methodology (equal); project administration (supporting); resources (supporting); supervision (supporting); writing – original draft (supporting); writing – review and editing (supporting). **Nosang V. Myung:** Conceptualization (equal); funding acquisition (equal); project administration (equal); resources (equal); supervision (equal); writing – original draft (supporting); writing – review and editing (supporting). **David M. Cwiertny:** Conceptualization (lead); funding acquisition (lead); investigation (supporting); methodology (lead); project administration (lead); resources (supporting); supervision (lead); writing – original draft (equal); writing – review and editing (equal).

## CONFLICT OF INTEREST

The authors report that they have no known competing financial interests or personal relationships that could have appeared to influence the work reported in this article.

## Supporting information


**Table S1.** Electrospun filters previously developed as alternative filters for PPE.
**Figure S1. (A)** Schematic diagram of the custom‐built electrospinning set‐up, consisting of a polymer precursor solution (sol–gel) in a syringe with a metal needle, a high‐voltage power supply, and grounded collector. **(B)** Picture of the electrospinning setup during the fabrication of a PS microfiber filter. Key parts of the set‐up are labeled.
**Table S2.** Electrospinning parameters for PS, 6% Ag/PS, and 25% Ag/PS fabrication.
**Figure S2.** Representative cross‐section images of PS nonwoven microfiber layers electrospun from 3.5 ml **(A)**, 4 ml **(B)**, and 4.5 ml **(C)** precursor solution volumes, as well as 6% Ag/PS **(D)** and 25% Ag/PS **(E)** nonwoven microfiber layers electrospun from 4 ml precursor solution volumes.
**Figure S3.** Filter media efficiency testing apparatus. Ports used to connect the upper and lower portion of the sample column (pass‐through cylinder) were also used to determine the pressure drop across the filter.
**Figure S4. (A)** Steps for one full repetition of the moderate handling procedure, shown on the PS_4.9_ filter material. As shown, the PS_4.9_ filter material is pliable and easily folded and twisted during handling. **(B)** Steps for the aggressive handling procedure (two repetitions are shown). **(C)** PS_4.9_, **(D)** 6 wt% Ag/PS, and **(E)** 25 wt% Ag/PS filter samples with no handling (i.e., pristine condition), after moderate handling, and after aggressive handling.
**Table S3.** Viscosity and electrical conductivity of precursor solutions for PS and Ag/PS microfiber materials.
**Figure S5.** ATR‐FTIR spectra of polystyrene microfibers fabricated herein via electrospinning. Aside from a feature (noted) due to adventitious carbon dioxide, features in the spectra are as expected based on previously reported reference IR spectra for polystyrene materials (Zolotarev 2017)
**Figure S6.** EDS images of 6 wt% Ag/PS **(A)** and 25 wt% Ag/PS **(B)** microfibers; the relatively bright spots (red) detected through back scattered electron (BSE) images were confirmed via EDS to be AgNPs on the surface of fibers. AgNPs were detected at higher density for materials with 25 wt% Ag (B) relative to those with 6 wt.% (A). Color SEM of 6wt% Ag/PS microfiber **(C)** also indicates the presence of AgNPs (shown in red) against the carbon of the polystyrene (shown in green); the nanoparticles embedded underneath the surface of the microfibers appear at a lower intensity of red.
**Figure S7.** Average solidity of PS microfiber material with respect to volume of sol gel used during electrospinning. All averages are within standard deviation of each other.
**Table S4.** Material strength testing results (mean and standard deviation of at least 5 replicates).
**Figure S8.** Example efficiency curve for media with no charge, illustrating how the mechanical forces, which impact particle deposition, influence the shape of the curve and the location of the minimum efficiency.
**Figure S9. (A)** Filtration efficiency and **(B)** pressure drop of PS_4.9_, 6 wt% Ag/PS, and 25 wt% Ag/PS filter materials after moderate and aggressive handling. Also provided for comparison are the filter efficiency (96.4%) and pressure drop (6.96 mmH_2_O) measured for an N95 FFR (dashed line in each panel), as well as results obtained with pristine (unhandled) filter materials. All three filter materials exhibit higher filtration efficiency after moderate handling, likely due to material compaction, while the pressure drop across the materials after moderate handling remain comparable or lower (in the case of the 25 wt% Ag/PS filter) to that measured for pristine materials. Similar results are seen for the PS_4.9_ and 6 wt% Ag/PS filters after aggressive handling and deterioration. The 25 wt% Ag/PS filter material exhibits a lower pressure drop after aggressive handling, while filtration efficiency is slightly decreased but still well above 95% after aggressive handling.
**Table S5.** Surface area coverage of AgNP (AgNP%) for 25 wt% Ag‐amended PS filters following handling procedures.
**Figure S10.** XRD patterns of 25 wt% Ag/PS filter material in pristine condition (no handling) and after moderate and aggressive handling procedures. The peaks located at the 2θ values of 38.49°, 44.73°, 64.91°, 77.88°, 82.07° are labeled with the corresponding Miller indices, which match the cubic crystalline planes of metallic Ag (Zhang et al. 2013). The XRD patterns demonstrate the strong presence of AgNPs in all three handled filters, consistent with no significant loss of AgNPs from the handling procedures (as suggested from complementary SEM analysis; see Table S4).

## Data Availability

The data that support the findings of this study are available from the corresponding author upon reasonable request.
