# Supplementary material for: Simple fabrication of an electrospun polystyrene microfiber filter that meets N95 filtering facepiece respirator filtration and breathability standards
Source: J Appl Polym Sci. 2022 Nov 21;140(5):e53406. doi: 10.1002/app.53406 (PMC10078598; doi:10.1002/app.53406)
Supplement: Supplementary file 1 — Table S1. Electrospun filters previously developed as alternative filters for PPE. Figure S1. (A) Schematic diagram of the custom‐built electrospinning set‐up, consisting of a polymer precursor solution (sol–gel) in a syringe with a metal needle, a high‐voltage power supply, and grounded collector. (B) Picture of the electrospinning setup during the fabrication of a PS microfiber filter. Key parts of the set‐up are labeled. Table S2. Electrospinning parameters for PS, 6% Ag/PS, and 25% Ag/PS fabrication. Figure S2. Representative cross‐section images of PS nonwoven microfiber layers electrospun from 3.5 ml (A), 4 ml (B), and 4.5 ml (C) precursor solution volumes, as well as 6% Ag/PS (D) and 25% Ag/PS (E) nonwoven microfiber layers electrospun from 4 ml precursor solution volumes. Figure S3. Filter media efficiency testing apparatus. Ports used to connect the upper and lower portion of the sample column (pass‐through cylinder) were also used to determine the pressure drop across the filter. Figure S4. (A) Steps for one full repetition of the moderate handling procedure, shown on the PS4.9 filter material. As shown, the PS4.9 filter material is pliable and easily folded and twisted during handling. (B) Steps for the aggressive handling procedure (two repetitions are shown). (C) PS4.9, (D) 6 wt% Ag/PS, and (E) 25 wt% Ag/PS filter samples with no handling (i.e., pristine condition), after moderate handling, and after aggressive handling. Table S3. Viscosity and electrical conductivity of precursor solutions for PS and Ag/PS microfiber materials. Figure S5. ATR‐FTIR spectra of polystyrene microfibers fabricated herein via electrospinning. Aside from a feature (noted) due to adventitious carbon dioxide, features in the spectra are as expected based on previously reported reference IR spectra for polystyrene materials (Zolotarev 2017) Figure S6. EDS images of 6 wt% Ag/PS (A) and 25 wt% Ag/PS (B) microfibers; the relatively bright spots (red) detected through back scattered [file APP-140-0-s001.docx]

Supporting Information

**Simple fabrication of an electrospun polystyrene microfiber filter that meets N95 FFR filtration and breathability standards**

Madeline G. Jensen^a^, Patrick T. O’Shaughnessy^b^, Marlee Shaffer^c^, Sooyoun Yu^d^, Yun Young Choi^e^[[1]](#footnote-2)^^, Megan Christiansen^f^, Charles O. Stanier^f^, Michael Hartley^g^, Joey Huddle^h^, Jed Johnson^h^, Kyle Bibby^c^, Nosang V. Myung^d^, and David M. Cwiertny^a,f^*

^a^Department of Civil and Environmental Engineering, University of Iowa, Iowa City, IA, United States; ^b^Department of Occupational and Environmental Health, University of Iowa, Iowa City, IA, United States; ^c^Department of Civil and Environmental Engineering and Earth Sciences, University of Notre Dame, Notre Dame, IN, United States; ^d^Department of Chemical and Biomolecular Engineering, University of Notre Dame, Notre Dame, IN, United States; ^e^Department of Chemical and Environmental Engineering, University of California Riverside, Riverside, CA, United States; ^f^Department of Chemical and Biochemical Engineering, University of Iowa, Iowa City, IA, United States; ^g^Department of Hospital Administration, University of Iowa Hospitals and Clinics, Iowa City, IA, United States; ^h^Nanofiber Solutions, LLC, Dublin, OH, United States

*CONTACT David M. Cwiertny david-cwiertny@uiowa.edu Department of Civil and Environmental Engineering, University of Iowa, 4655 Seamans Center for the Engineering Arts and Sciences, Iowa City, IA, 52242, United States.

Prepared for Journal of Applied Polymer Science

November 11, 2022

1. **SUPPLEMENTAL METHODS**

**Microfiber Filter Fabrication.** Filter materials were fabricated using a custom-built electrospinning system described in previous work (Peter et al. 2016) and shown in **Figure S1**. Fabrication parameters are summarized in **Table S2**.

**Characterization.** *Precursor Solution Properties.* Viscosity and electrical conductivity of electrospinning precursor solutions were measured using a viscometer (Brookfield DV-I Prime) and a glass-body electrical conductivity probe (K=0.1, Oakton) paired with an embedded conductivity circuit (Atlas Scientific, EZO-EC™) and an Arduino Uno Rev3 board, respectively. Viscosity and electrical conductivity measurements of the electrospinning sol gel solutions can be found in **Table S3**.

*Fiber Morphology.* Morphology of the fibers was characterized by Scanning Electron Microscopy (SEM, ThermoFisher Prisma E) at 10-20 kV. Both secondary electron and backscatter electron images were obtained. Samples were coated with a thin layer of gold using a sputter coater (Electron Microscopy Sciences 575X) at 20 mA for 30 seconds to minimize charging before taking SEM images. Elemental analysis of the nanofibers was performed on the same SEM using the Energy Dispersive X-ray Spectroscopy (EDS) function.

*Physical Properties*. Cross-section images (**Figure S2**) were taken via a 50-1000x digital microscope (JALIELL Wireless Digital Microscope) and analyzed using ImageJ software to determine material thickness. To account for variability in thickness across the filter layer, cross-sections were taken of the entire filter width, with thickness measurements taken every 0.5 cm. Cross-sections of three to four replicate batches were analyzed for each filter material type. For filter thickness measurements, we report averages and standard deviations calculated from the collective measurements of these replicate batches. Area and mass measurements were collected and averaged for each filter material type, with three to six replicate batches analyzed per filter material type. Average volumes of the filter materials were then calculated from the average layer thickness and area values, with standard deviations determined through error propagation. Likewise, average densities of the filter materials were calculated based on the average mass and volume values, with standard deviations determined through error propagation. Solidity— or packing density— of the filter materials was then determined by dividing the average density of the filter by the densities of the base materials (PS and AgNPs, if applicable). The PS density value used for these calculations was 1.047 g/cm^3^, as reported by the vendor Sigma-Aldrich. The Ag nanoparticle density value used was 10.49 g/cm^3^, as reported by the vendor Nanostructured & Amorphous Materials, Inc. The mass percentages of PS and AgNP in the end product were taken into account during these calculations.

*Tensile Strength Testing*. Test samples were cut into a dog-bone shape with gauge width of 3.18mm (ASTM Die 638 Type V). The thickness of each sample’s gauge region was measured and recorded for calculation of the ultimate tensile strength. Samples were inspected to ensure the gauge regions were free of notches or other defects. A grip was mounted to the base of the Universal Test Machine (Mecmesin, Sterling, VA). A second grip was mounted to the load cell on the mobile arm of the Universal Test Machine and set to travel at a rate of 25mm/min. A data logger was set to take force and displacement/position readings at a constant rate. The sample was secured in the grips, and slack was minimized. The load frame was zeroed, and sample was strained until failure. The peak load of each sample was recorded, the strain (% elongation at break) and the ultimate tensile strength (UTS) was calculated. UTS calculations were performed using Equation S-1. Results can be found in **Table S-3**.

$UTS \left( MPa \right)=\frac{Peak Load (N)}{\left[ Gauge Width \left( mm \right) \times Sample Thickness (mm \right]}$ (S-1)

**Filter Efficiency and Pressure Drop Testing.** *Filter Efficiency.* Particle capture efficiency testing followed the procedure described in 42 CFR 84.181 and as described in a NIOSH test standard for certifying N95 FFRs (NIOSH 2019). A full description of the apparatus is provided by O’Shaughnessy et al. (2021). A schematic of the apparatus is provided in **Figure S3**. Briefly, an NaCl aerosol generated with a nebulizer from a 2% NaCl solution was dried, neutralized, and diluted with filtered air to produce particle-laden air at 25°C (77°F) and 35% RH. The particle-laden air was then drawn through a column exposing a 27-mm diameter portion of filter media sample clamped tightly between an upper and lower section of the column (Figure S3). The airflow required by the NIOSH N95 efficiency test is 85 L/min through an entire FFR. Here, with only 27 mm diameter of sample exposed in the column, the airflow rate through the column was adjusted so that the velocity of air drawn through the filter sample (face velocity) of 8 cm/s was equivalent to the face velocity of an entire typical FFR at 85 L/min. Airflow calibration was conducted using a primary calibrator (Gilian Gilibrator 2, Sensidyne, Clearwater, FL) before each test. The aerosol particle count and size distribution were measured with a scanning mobility particle sizer (SMPS) consisting of an electrostatic classifier (Model 3080, TSI Inc., Shoreview, MN) combined with a condensation particle counter (CPC) (Model 3785, TSI Inc., Shoreview, MN). As configured, the SMPS measured particles in 103 size bins between 7.4 – 290 nm, which encompassed the distribution of salt aerosol diameters. Particle sampling occurred both upstream and downstream of the filter media. Particle capture efficiency (filter efficiency, *E*) was then determined as a ratio of the total particle concentration downstream (*C_d_*) and upstream (*C_u_*) of the filter media over the entire range of the NaCl particle distribution (Equation S-2):

$E\left( \% \right)=\left( 1-\frac{C_{d}}{C_{u}} \right)100$ (S-2)

*Pressure Drop Testing.* Pressure drop testing of materials for this study generally followed the procedure described in 42 CFR 84.180. Filter pressure drop measurements were conducted using the sampling column used for the efficiency testing. The same ports used to sample above and below the filter media were connected to a sensitive, calibrated pressure transmitter (Series 646, Dwyer Instruments, Inc., Michigan City, MI) that measures air pressure in the range of 0 – 65 mm H_2_O. The voltage output signal of the transmitter was received by an analog-to-digital converter and read using the LabVIEW software system (National Instruments, Austin, TX). During a pressure trial, the media was subjected to the same flow rate applied during particle capture efficiency testing to achieve 8 cm/s face velocity. It should be noted that pressure drop measurements were made before efficiency measurements, so the values reflect the pressure drop of the filter media before filtration of NaCl particles.

**Material Disinfection**. Material samples were disinfected via the decontamination process used at the University of Iowa Hospital and Clinics (UIHC) for decontaminating N95 FFRs (Cramer et al. 2021; Welch et al. 2021). Material samples were disinfected using a SteraMist™ Surface Unit (TOMI Environmental Solutions, Inc., Frederick, MD). Originally developed as a countermeasure for biological warfare, this process ionizes a stream of 7.8% hydrogen peroxide (H_2_O_2_) using a 17,000 V cold plasma arc to produce a fine mist of ionized hydrogen peroxide (iHP) (TOMI Environmental Solutions n.d.). The iHP mist was sprayed directly onto material samples from a distance of 24 inches (61 cm) at one-second-per-side intervals. For a full disinfection cycle, samples were sprayed 3 times each side for a total iHP exposure of 6 seconds, then left to dry. Samples underwent one and six full disinfection cycles, then were tested for their filter efficiency and pressure drop performance afterwards.

**Antimicrobial Testing**. Assay methods analyzed direct bacterial contact with the material. These methods were adapted from Huang et al. (2020). A solution using 25 mL of Luria-Bertani (LB) Broth and 60 µL of *E. coli* was combined and left in a shaking incubator (150 rpm) at 37 ºC overnight, as per EPA Methods 1602 (USEPA Office of Science 2001), with growth confirmed by optical density measurements. Each of the developed filter materials, as well as a standard surgical mask as a control, were cut into 1-cm squares. Three squares of each material type were spotted with 100 µL of broth and were left to dry for 36 hours. Each filter square was placed in a test tube with 1.3 mL of 1X PBS ensuring the materials were fully submerged and were incubated for 1 hour at 37º C. The tubes were vortexed for 45 seconds at 2000 rpm to remove any bacteria on the surface of the material. Serial dilutions were produced for each material and 100 microliters of each dilution was plated on mTEC agar. Triplicates of each dilution were plated and incubated for 18 hours at 37 ºC before they were removed and counted for colony forming units (CFU). Blank plates were also incubated for quality control to ensure that contamination did not occur, and no control indicated contamination. The antimicrobial capabilities of each material were then determined via logarithmic reduction of triplicate average CFU counts, in which the log of the material’s average CFU was subtracted from the log of initial CFU in the broth.

**Microfiber Filter Handling Simulation.** To simulate their durability during use, PS_4.9_, 6 wt% Ag/PS, and 25 wt% Ag/PS filter materials were subjected to two simulated levels of handling: “moderate” (typical handling and use) and “aggressive” (rough handling and overuse). For “moderate” handling, filter materials were folded in half twice, unfolded, twisted, and then untwisted for one full repetition (**Figure S4-A**); this was repeated for a total of fifty repetitions. For “aggressive” handling, filter materials were crumpled with simultaneous abrasive rubbing, then uncrumpled, a total of fifty repetitions (**Figure S4-B**).

**AgNP Retention Evaluation**. For 25 wt% Ag-amended filters, the retention of Ag particles within the PS fibers was assessed using SEM imaging to compare the surface area coverage of AgNPs before and after both handling procedures. Specifically, Ag-modified fibers were imaged via SEM with concentric backscatter (CBS) detection, where AgNPs were identifiable via their bright contrast with the surrounding PS fibers. ImageJ was used to estimate the total area of the imaged fibers as well as the area attributable to the AgNPs on the fiber surface. The surface area coverage of AgNPs was then calculated for each sample as a percentage, determined from the total AgNP surface area normalized to the total available area of the Ag-modified PS fiber (Equation S-3).

$AgNP\%=\frac{\left[ Total surface area of AgNP \right]}{\left[ Total available surface area of the Ag-modified fiber \right]}$ (S-3)

Fifty to one hundred fibers were analyzed per handled sample (pristine, moderate, and aggressive), with averages and standard deviations calculated from the collective fiber measurements. Results can be found in **Table S5**. In complement to the SEM analysis, XRD (Rigaku MiniFlex600) characterization revealed the expected diffraction pattern for metallic Ag in the 25 wt% Ag-amended material before and after both handling procedures; the results can be found in **Figure S9**.

**Filter Modeling.** A mathematical model of filter performance was created in Microsoft Excel. The primary sources consulted for its development were the texts by Hinds (1999) and Brown (1993), as well as the papers by Balazy et al. (2006) and Chen, Lehtimäki, and Willeke (1993). Essentially, equations were applied to the model that take into account the forces of diffusion, interception, impaction, and gravity on the efficiency of a single fiber of a given diameter. The efficiency of a combination of these fibers of a given depth and solidity (the inverse of porosity) is then calculated for a given face velocity. Although a model of filtration performance can also include equations to include the application of electrostatic forces caused by “electret” filter media, this model was developed without that force applied under the assumption that the electrospun filter media described here was not charged. **Figure S7** provides plots of the filter efficiency resulting from each of the forces modeled, which displays the characteristic dip in efficiency where diffusion begins to diminish, and the inertial-based forces of impaction and interception have not reached their full effect. This results in a minimum efficiency at some particle size referred to as the most penetrating particle size (MPPS). Here that minimum is shown to be near 0.2 μm for an uncharged filter that has a minimum efficiency > 90%.

The spreadsheet also included a model of pressure drop from Hinds (1999, Eq. 9.36), which was adapted from Davies (1953) and has been found to be acceptably accurate when predicting filter pressure drop for filter media with solidities < 0.3 and low Knudsen numbers determined from fiber diameter (< 0.25) (Maze et al. 2007). Therefore, both efficiency and pressure drop could be assessed while changing filter properties (e.g., fiber diameter, filter solidity, and filter depth) and maintaining face velocity at 8 cm/s. This analysis indicated that a combination of microfibers (5 – 10 μm) with corresponding Knudsen numbers in the range of 0.027 – 0.013, a filter depth of 3 – 5 mm, and a solidity near 0.1 would result in efficiencies > 90% while minimizing pressure drop; parameters that were then used as the basis for filter media fabrication.

1. **SUPPLEMENTAL FIGURES AND DATA**

**Table S1.** Electrospun filters previously developed as alternative filters for PPE.

| Reference | Material | Fabrication Method | Summary of Results |
| --- | --- | --- | --- |
| SUBSTRATE-SUPPORTED MATERIALS | | | |
| Akduman 2019 | Cellulose acetate (15-16 w/v%) and PVDF (10,12,14 w/w%) nanofiber layer on PP spunbond substrate | Single-needle electrospinning onto PP spunbond substrate | Best variations met 95% FE with PD near 30 mm H_2_O |
| Ruan et al. 2020 | Polyacrylonitrile:TiO_2_ and polyacrylonitrile- co-polymethyl acrylate:TiO2 nanofiber layers on PP nonwoven support substrate | Single-needle electrospinning onto PP nonwoven substrate; TiO_2_ integrated into precursor solution | 90-100% FE, with PD around 160 mm H_2_O; FE was lower for smaller (0.3-1µm) particles vs larger (1-3 µm) particles in general |
| Kim et al. 2017 | PVDF nanofibers with Fe_3_O_4_ NPs (5, 10, 20 wt%) on metallic screen filter support | Single-needle electrospinning onto metallic screen filter; Fe_3_O_4_ NPs integrated into precursor solution | 73-97% FE for 0.3 µm dust particles, with 1.75-2.75 mm H_2_O PD; FE improves & PD lowers with increasing wt% of Fe_3_O_4_ NPs |
| He et al. 2021 | 5-bromosalicylic acid (1 wt%) /polyvinyl butyral (7-10 wt%) nanofibers on nonwoven fabric | Single-needle electrospinning onto nonwoven substrate; 5-bromosalicylic acid integrated into precursor solution | 91-99% FE, with 10-22 mm H_2_O PD for 0.3 µm salt particles; 5-log removal of *E. coli* & *S. aureus* |
| Leung and Sun 2020 | Charged PVDF nanofibers on PP substrate (monolayered & multi-layered) | Single-needle electrospinning onto PP substrate; fiber material then charged with corona discharge | Filters consisting of smaller diameters with smaller basis weight per layers and more layers, or larger diameters with larger basis weight per layer and fewer layers, met at least 90% FE for 100 nm aerosol salt particles with <30 mm H_2_O PD |
| Kang et al. 2021 | PVDF (13-15%) nanofibers on electrified melt-blown PP nonwoven fabric | Multi-needle electrospinning onto PP nonwoven substrate | All filter variations met ≥95% FE for aerosolized salt particles, with 8-16 mm H_2_O PD; the thin nanofiber layer addition increased FE of the PP nonwoven by 15-19% |
| Saikaew and Intasanta 2021 | Polyacrylonitrile/PVDF nanofibers with mangosteen extract and Ag NPs onto nonwoven substrate | Electrospinning (single needle up to industrial double-walled multi-needle) onto nonwoven substrate; polymer solutions were created separately then mixed together, followed by addition of mangosteen extract and AgNO_3_ to solution | PAN/PVDF/GM/Ag filter had 93.91% FE for 0.5 µm particles, with PD of 8.3 mm H_2_O/cm^2^; antimicrobial abilities against S. aureus and K. pneumoniae by direct contact, and M. smegmatis and M. tuberculosis by modified direct contact; antiviral activity (> 80%) against H1N1 influenza virus |
| Zhang, Tang, et al. 2016 | Polysulfone (microfiber) /polyacrylonitrile (nanofiber) /polyamide-6 (nanonets) layered filter on nonwoven substrate | Multi-needle electrospinning onto nonwoven PTE substrate; polysulfone, polyacrylonitrile, and polyamide-6 layers were spun sequentially onto substrate; treated with solvent immersion to remove charges | FE and PD simulated based on layer and fiber characteristics; the three layers of filter could filter out particles from 0.3-2 µm particles with a PD of ~12 mm H_2_O |
| Zhang, Liu, et al. 2016 | poly(ethylene oxide)@polyacrylonitrile/polysulfone (PEO@PAN/PSU) composite membranes on nonwoven PP substrate | Multi-needle electrospinning; PEO/PAN and PSU solutions were made separately, then electrospun simultaneously at different ratios; membranes vacuum-dried at 100 °C for 1 hr to form bonding structures from PEO solidification | Best variation displayed 99% FE for 300-500 nm aerosolized salt particles with ~10 mmH_2_O PD; bonding points via PEO prevented structural collapse of filter under stress |
| Li et al. 2020 | Polystyrene /PVDF electret filter on polyethylene nonwoven substrate | Single-needle electrospinning onto polyethylene nonwoven substrate; electret properties were endowed in situ during electrospinning process | Hybrid PS/PVDF fibers showed enhance electret effect; best PS/PVDF variation achieved 84% FE with low PD (< 1 mm H_2_O) |
| Pais et al. 2021 | Polyamide nanofibers with PP microfibers | Single-needle electrospinning onto PP microfiber substrate; another PP microfiber layer was added on top of polyamide nanofibers to form multilayer system | Optimal multilayer system had FE of 94.1% for aerosolized salt particles, and ~23 mm H_2_O PD for a face velocity of 95 L/min |
| MORE COMPLEX FABRICATION PROCESSES | | | |
| Choi et al. 2021 | poly(butylene succinate) nanofiber/microfiber Janus membrane filter coated with chitosan whiskers | Single-needle electrospinning of fibers onto water bath surface, which are then transferred to PET frame. Chitin was hydrolyzed, deacetylated, neutralized, freeze-dried, then dispersed into water. Fiber layers were then dipped into the chitosan whisker solution to coat. | ~98% FE of 2.5 µm particles with ~6 mm H_2_O PD; nanofibers and chitosan whiskers filtered out particles through physical sieving and electrostatic adsorption, microfibers reduced PD and supported the chitosan nanowhiskers |
| Kao et al. 2016 | Aligned polyacrylonitrile nanofiber microscaffolds | Single-needle electrospinning; fiber layers were pre-aligned via rotation rate of controller, then further aligned through DMF vapor annealing; microscaffolds were created layer by layer with different fiber orientations | 9-layer square oriented microscaffold had 99.997% FE of ≥ 0.1 µm particles, with 5.6 mm H_2_O PD |
| Jung et al. 2019 | Polystyrene fibers with modified surface energy | Single- needle electrospinning; fibers were then modified via O_2_ plasma treatment (to increase surface energy) or C_4_F_8_ plasma enhanced chemical vapor deposition | Unmodified and modified PS filters had low PD (6.4-6.8 mm H_2_O); modification lowered FE of the PS filter from 86.5% to 72% and 31.4% for NaCl aerosol particles; modification did not improve oily aerosol or bacterial filtration |
| Reyes and Frey 2017 | Nylon-6 (grafted w/ acrylic acid) | Multi-nozzle large scale electrospinning; grafted w/ acrylic acid and gamma irradiation | 60-95% FE of 0.2-6 µm aerosolized salt particle  Non-grafted materials performed better than grafted materials by ~20% |
| ANTIBACTERIAL MATERIALS | | | |
| Z. Zhu et al. 2021 | UiO-PQDMAEMA/  Polyacrylonitrile filter | Single-needle electrospinning onto stainless steel mesh; 2-(dimethyl decyl ammonium) ethyl methacrylate (QDMAEMA) was polymerized and grafted on the surface of UiO-66-NH2 MOF in a 2-step process to obtain UiO-PQDMAEMA. UiO-PQDMAEMA was integrated into precursor solution with polyacrylonitrile | Met >95% FE for MPPS of 80 nm aerosolized particle at ~ 5 mm H_2_O PD; antimicrobial activity against both *S. epidermidis* (Gram-positive) and *E. coli* (Gram-negative) |
| Huang et al. 2019 | Polyacrylonitrile (5,8,10%)/ 1-chloro-2, 2, 5, 5-tetramethyl- 4-imidazolidinone (5%) nanofiber filter | Single-needle electrospinning | Material displayed good air permeability (27.3 mm/s); Material obtained 6-log removal within 1 min of contact for *S. aureus*, and 6.6-log removal after 10 min of contact with *E. coli* |
| Deng et al. 2022 | CNWs-CI-PTB-PHMG/NEO filter material | Pre-made cellulose nonwoven fabric (CNWs) were grafted with cyclohexyl isocyanate (CI), then grafted with poly(thiiran-2-yl methyl methacrylate-2-(4-benzoyl phenoxy)ethyl methacrylate) (PTB) via UV irradiation, the grafting of antiviral agents neomycin sulfate (NEO) and Polyhexamethyleneguanidine (PHMG) | High FE (~100%) with low PD (8-12 mm H_2_O) of aerosolized salt particles; higher antibacterial activity for *S. aureus* (Gram positive; ≥98%) than *E. coli* (Gram negative, 45-71%) over 10 minutes; high antiviral activity (≥99%) against SARS-CoV-2 |
| Vanangamudi, Hamzah, and Singh 2015 | PVDF (15 wt%)–Ag–Al_2_O_3_ (2,4,6,8 wt%) nanofibers | Single-needle electrospinning; AgNO_3_ and DMF was used to make Ag NPs, which were then added to PVDF precursor solution followed by Al_2_O_3_; after electrospinning membranes were heat treated and treated with UV for 20 minutes each | Materials held high FE (94-99%) of 0.36 µm oil particles, increasing with Al_2_O_3_ %; PD also increased with increasing Al_2_O_3_ %; antibacterial activity for *E. coli* was above 99% for all materials |
| Wang et al. 2019 | Polyacrylonitrile/AG NPs (!5 or 3%) nanofiber membrane | Single-needle electrospinning; AgNO_3_ and NaBH_4_ were reacted to form AgNPs, which were then added to PAN precursor solution | FE of 94%, 89%, and 82% for 10µm, 2.5 µm, and 1 µm particles for 0.5 m/s velocity; 3% Ag NP/PAN membrane had highest antimicrobial activity with 4-log removal for *E. coli* and *S. aureus* |
| Swamidoss et al. 2018 | Ag NP/ PVDF nanofibers on PP nonwoven substrate | Single-needle electrospinning onto PP nonwoven substrate; AgNO_3_ (5, 10, 15 wt%) was integrated into PVDF precursor solution, and mixed for 24 hrs until AgNPs formed in solution | No FE or PD data; materials reached ~99.9% bacterial efficiency for larger particle sizes (3-8 µm) |
| M. Zhu et al. 2018 | polyvinyl alcohol (PVA)/citric  acid (CA) electrospun nanofibrous membranes with SiO_2_ and Ag NPs | Single-needle electrospinning of PVA/CA (0.6 wt%); SiO_2_ NPs (0,2,4,6,8 wt%) and Ag NPs (0.1-0.5 wt%) were added to precursor solution before electrospinning; PVA /CA fibers were then thermally crosslinked. | PVA-CA- 4 wt% SiO_2_ membrane had highest FE (~90%) for 300-500nm particles, with ~ 4 mm H2O PD; antibacterial activity increased with increasing AgNP loading; 0.3 wt% AgNP loaded membranes at 4.82 g/m^2^ basis weight showed 99.8% FE with ~30 mmH_2_O |

FE= filter efficiency; PD= pressure drop (breathing resistance); PP= polypropylene; PVDF= polyvinylidene fluoride; NP= nanoparticles

**A)**


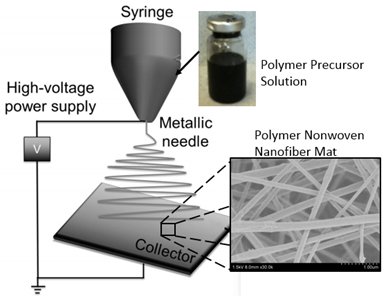

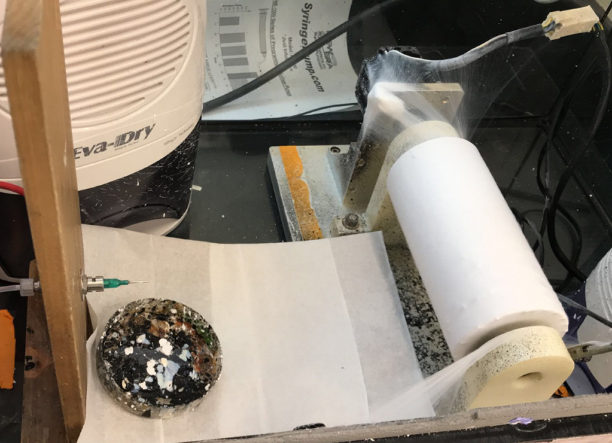


**B)**

Metallic needle with precursor solution

Collector with PS microfiber material

**Figure S1**. **(A)** Schematic diagram of the custom-built electrospinning set-up, consisting of a polymer precursor solution (sol-gel) in a syringe with a metal needle, a high-voltage power supply, and grounded collector. **(B)** Picture of the electrospinning setup during the fabrication of a PS microfiber filter. Key parts of the set-up are labeled.

**Table S2.** Electrospinning parameters for PS, 6%Ag/PS, and 25%Ag/PS fabrication.

| Syringe Pump Flow (ml/hr) | Distance needle-to-collector (cm) | Needle Gauge Size | Collector Rotation (rpm) | Voltage (kV) | E-spin Box Humidity (%) | E-spin Box Temp. (°F) |
| --- | --- | --- | --- | --- | --- | --- |
| 1.2 | 20 | 18G | 500-600 | 29 | 37-40 | 73-75 |

**
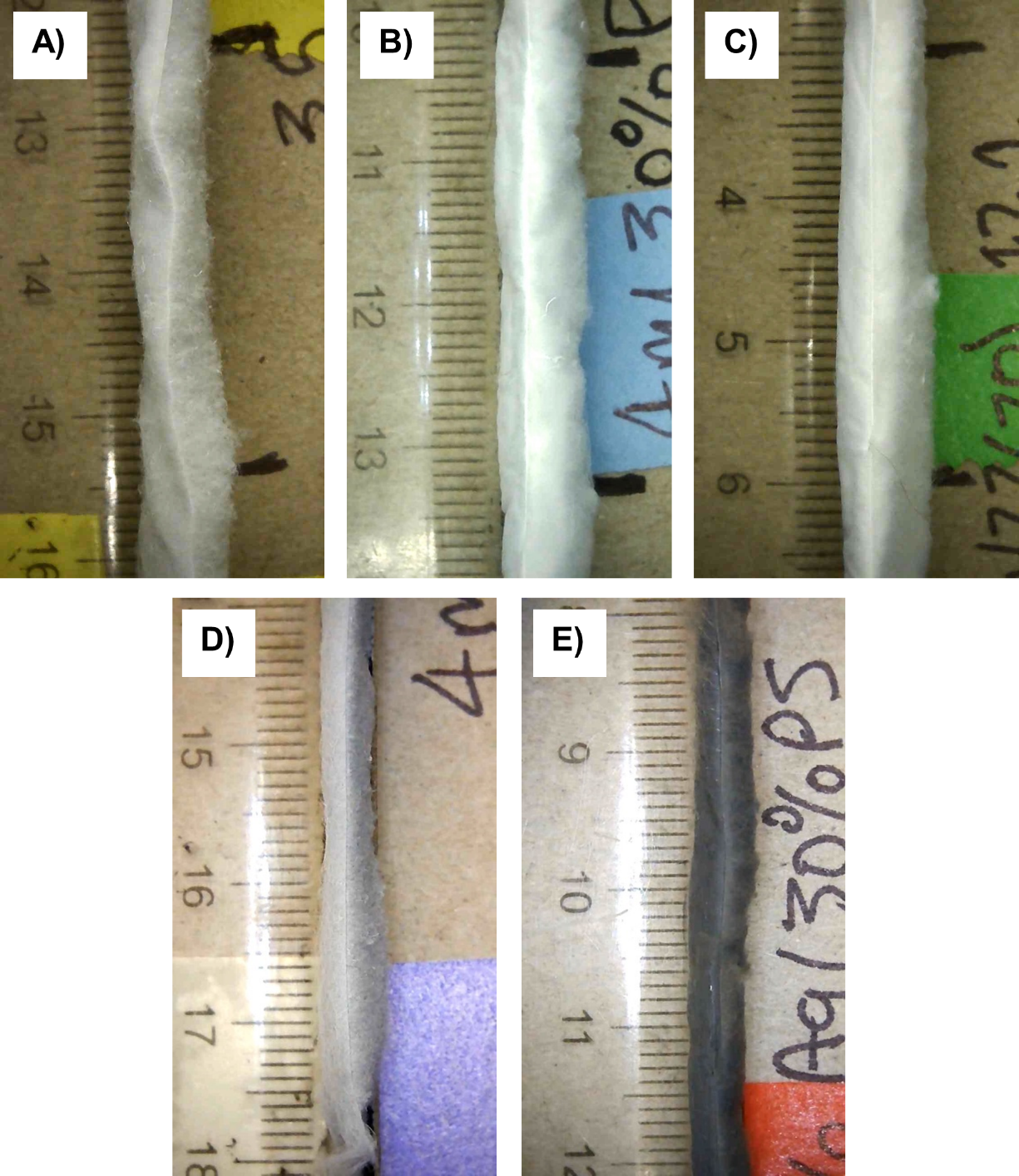
**

**Figure S2.** Representative cross-section images of PS nonwoven microfiber layers electrospun from 3.5 mL **(A)**, 4 mL **(B)**, and 4.5 mL **(C)** precursor solution volumes, as well as 6%Ag/PS **(D)** and 25%Ag/PS **(E)** nonwoven microfiber layers electrospun from 4 mL precursor solution volumes.


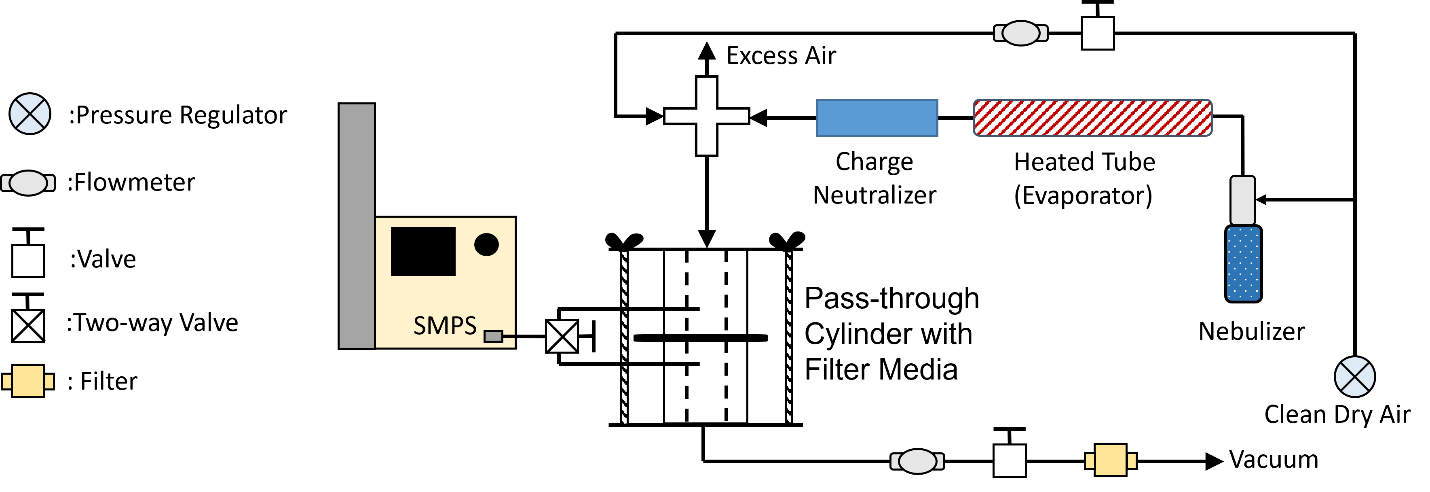


**Figure S3.** Filter media efficiency testing apparatus. Ports used to connect the upper and lower portion of the sample column (pass-through cylinder) were also used to determine the pressure drop across the filter.


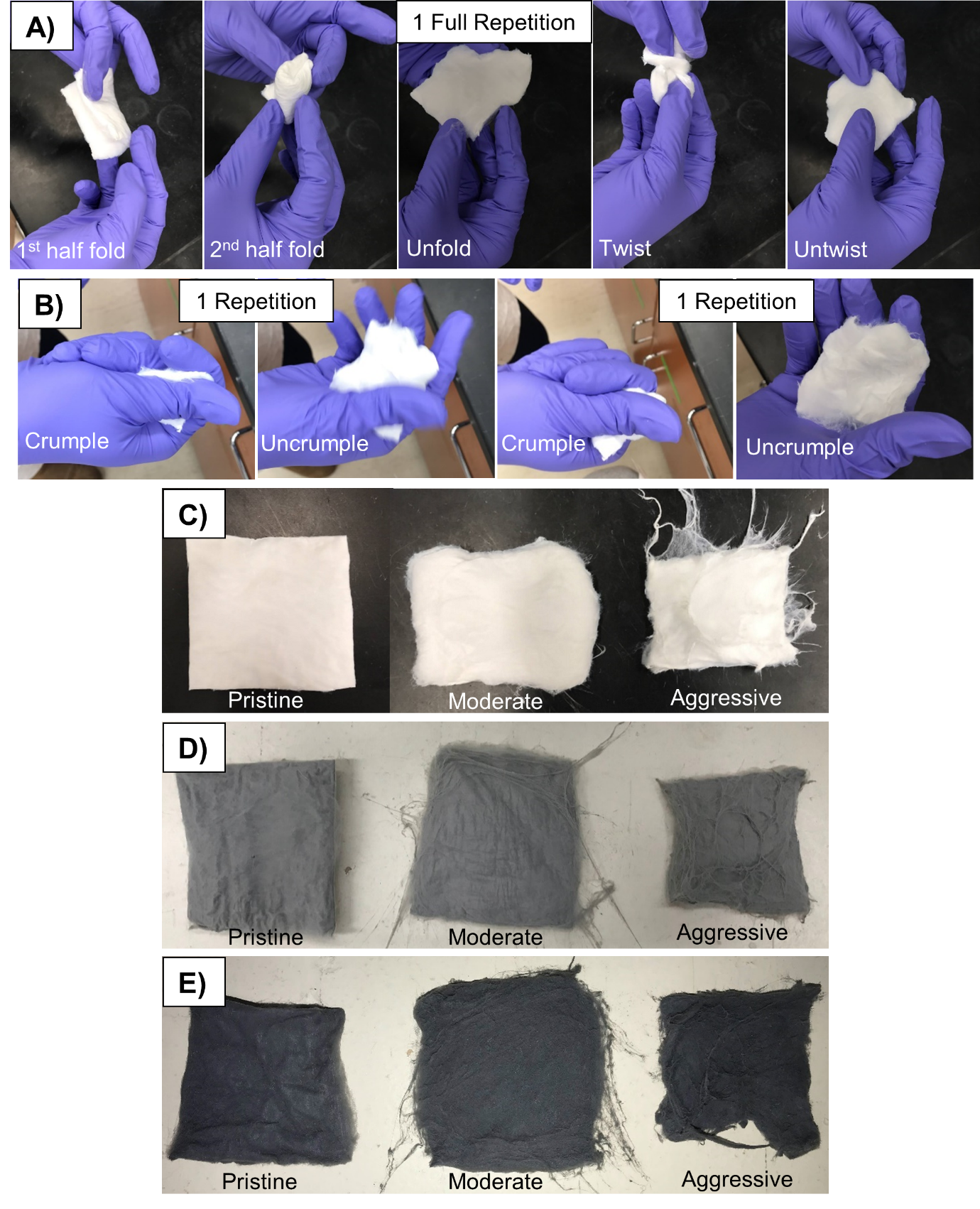


**Figure S4**. **(A)** Steps for one full repetition of the moderate handling procedure, shown on the PS_4.9_ filter material. As shown, the PS_4.9_ filter material is pliable and easily folded and twisted during handling. **(B)** Steps for the aggressive handling procedure (two repetitions are shown). **(C)** PS_4.9_, **(D)** 6 wt% Ag/PS, and **(E)** 25 wt% Ag/PS filter samples with no handling (i.e., pristine condition), after moderate handling, and after aggressive handling.

**Table S3.** Viscosity and electrical conductivity of precursor solutions for PS and Ag/PS microfiber materials.

| Solution conditions | Viscosity @25°C (cP) | Electrical Conductivity (µS/cm) |
| --- | --- | --- |
| PS | 2336 | 0.96 ± 0.17 |
| 6 wt% Ag/PS | 1899 | 7.08 ± 0.41 |
| 25 wt% Ag/PS | 2494 | 19.27 ± 0.14 |

**
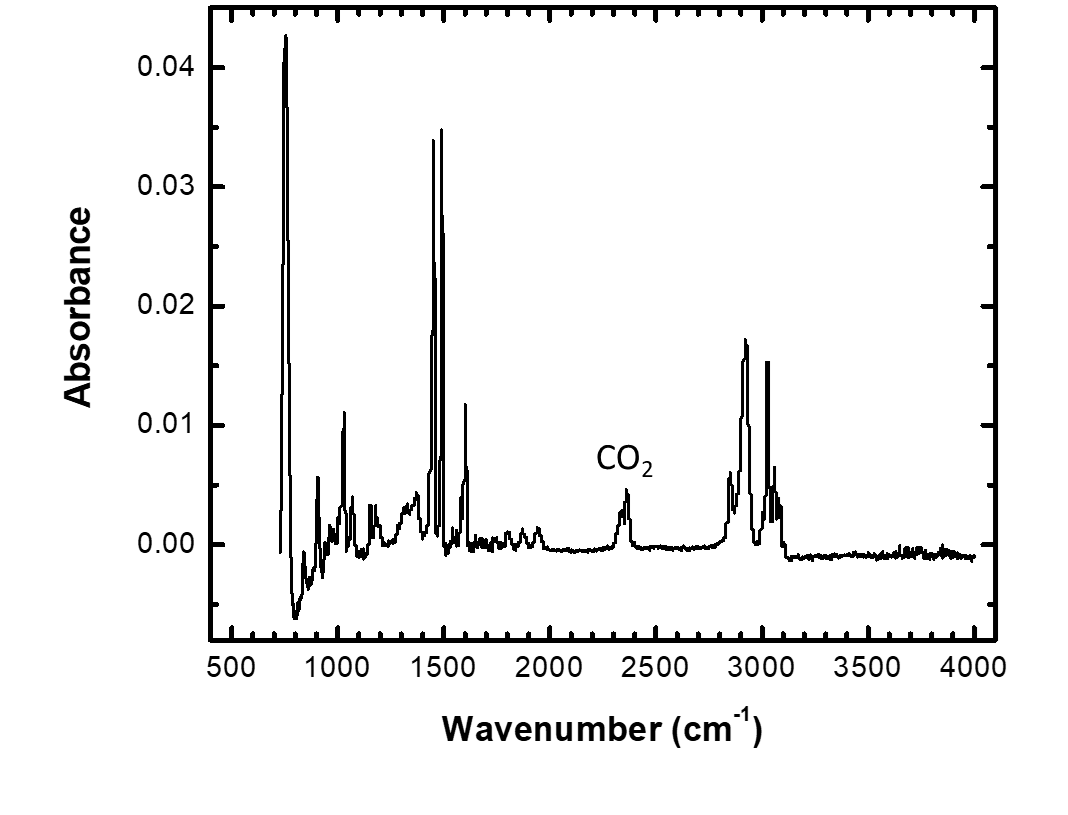
**

**Figure S5**. ATR-FTIR spectra of polystyrene microfibers fabricated herein via electrospinning. Aside from a feature (noted) due to adventitious carbon dioxide, features in the spectra are as expected based on previously reported reference IR spectra for polystyrene materials (Zolotarev 2017)


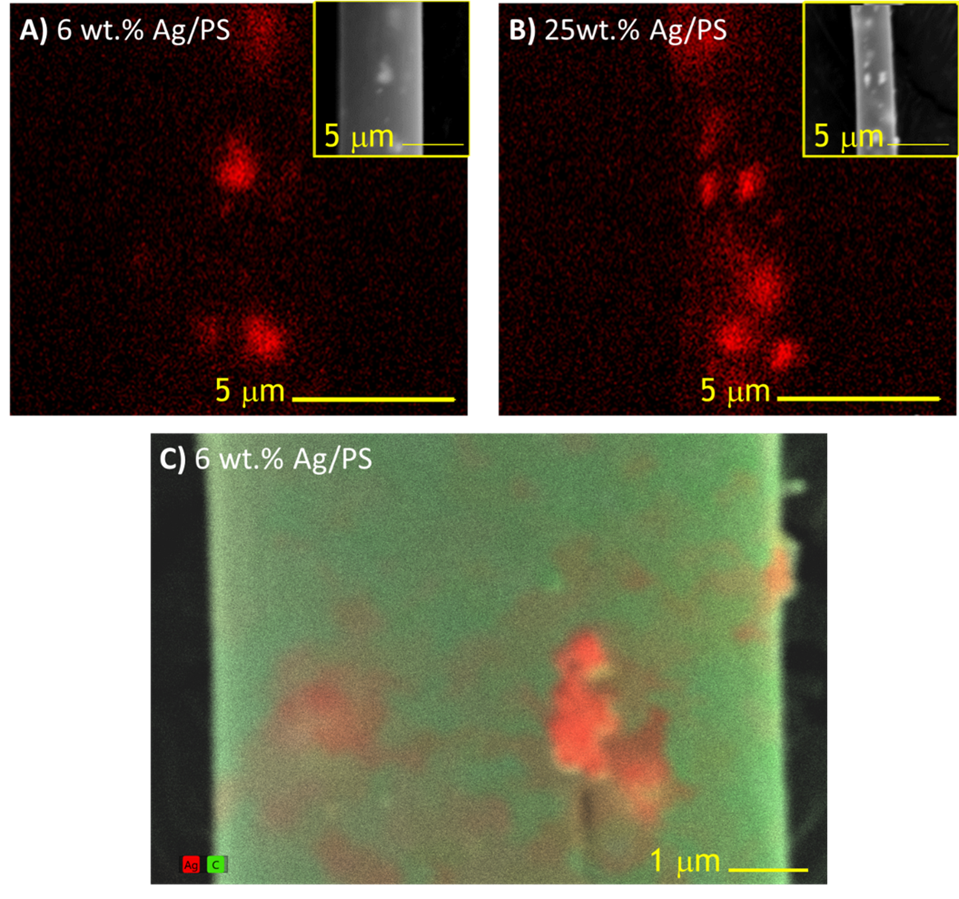


**Figure S6**. EDS images of 6 wt% Ag/PS **(A)** and 25 wt% Ag/PS **(B)** microfibers; the relatively bright spots (red) detected through back scattered electron (BSE) images were confirmed via EDS to be AgNPs on the surface of fibers. AgNPs were detected at higher density for materials with 25 wt% Ag (B) relative to those with 6 wt.% (A). Color SEM of 6wt%Ag/PS microfiber **(C)** also indicates the presence of AgNPs (shown in red) against the carbon of the polystyrene (shown in green); the nanoparticles embedded underneath the surface of the microfibers appear at a lower intensity of red.


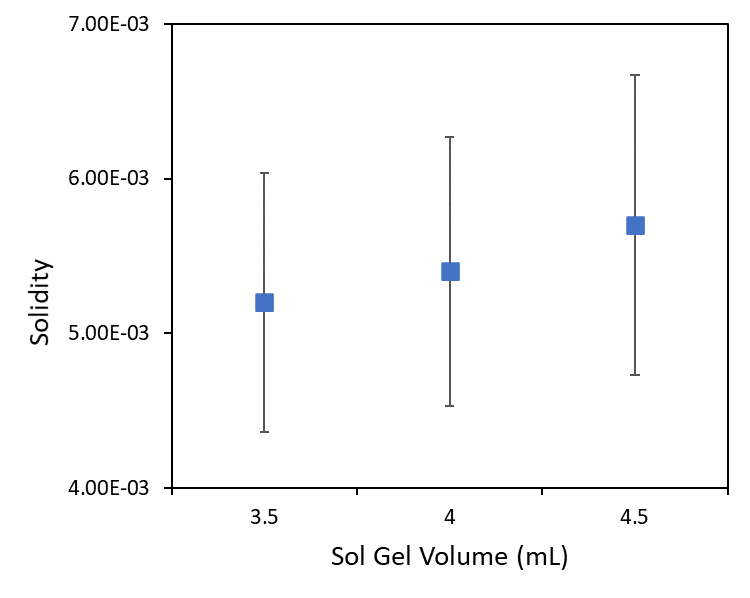


**Figure S7.** Average solidity of PS microfiber material with respect to volume of sol gel used during electrospinning. All averages are within standard deviation of each other.

**Table S4.** Material strength testing results (mean and standard deviation of at least 5 replicates).

| Filter Material | Ultimate Tensile Strength (MPa) | Elongation (%) | Modulus (MPa) |
| --- | --- | --- | --- |
| N95 Control | 0.556 ± 0.036 | 86.640 ± 25.169 | 3.321 ± 0.436 |
| PS_4.4_ | 0.744 ± 0.382 | 141.322 ± 86.863 | 1.005 ± 0.639 |
| PS_4.9_ | 0.547 ± 0.010 | 106.418 ± 82.232 | 0.906 ± 0.233 |
| PS_5.6_ | 0.544 ± 0.132 | 193.765 ± 55.140 | 0.819 ± 0.428 |


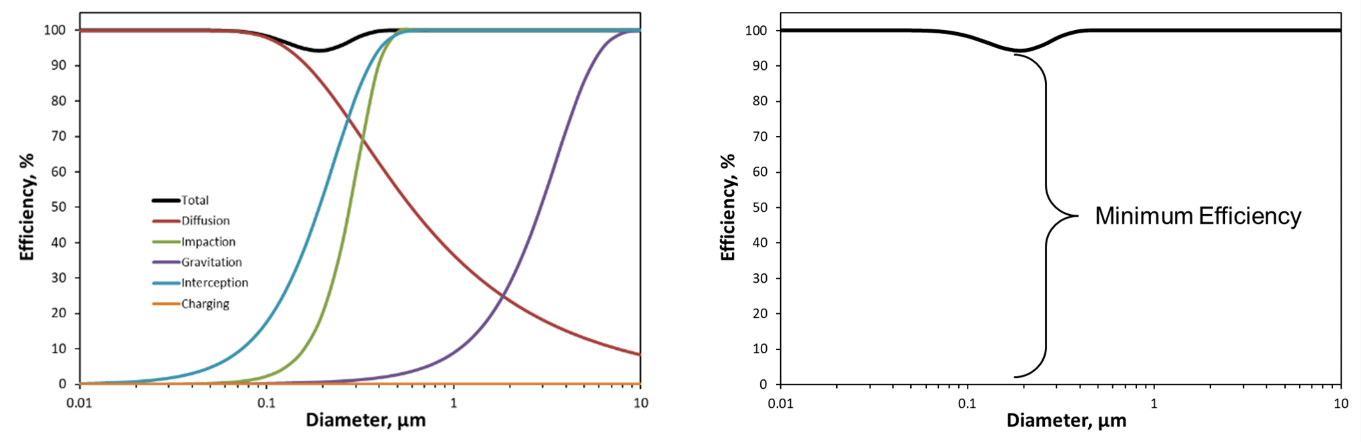


**Figure S8.** Example efficiency curve for media with no charge, illustrating how the mechanical forces, which impact particle deposition, influence the shape of the curve and the location of the minimum efficiency.


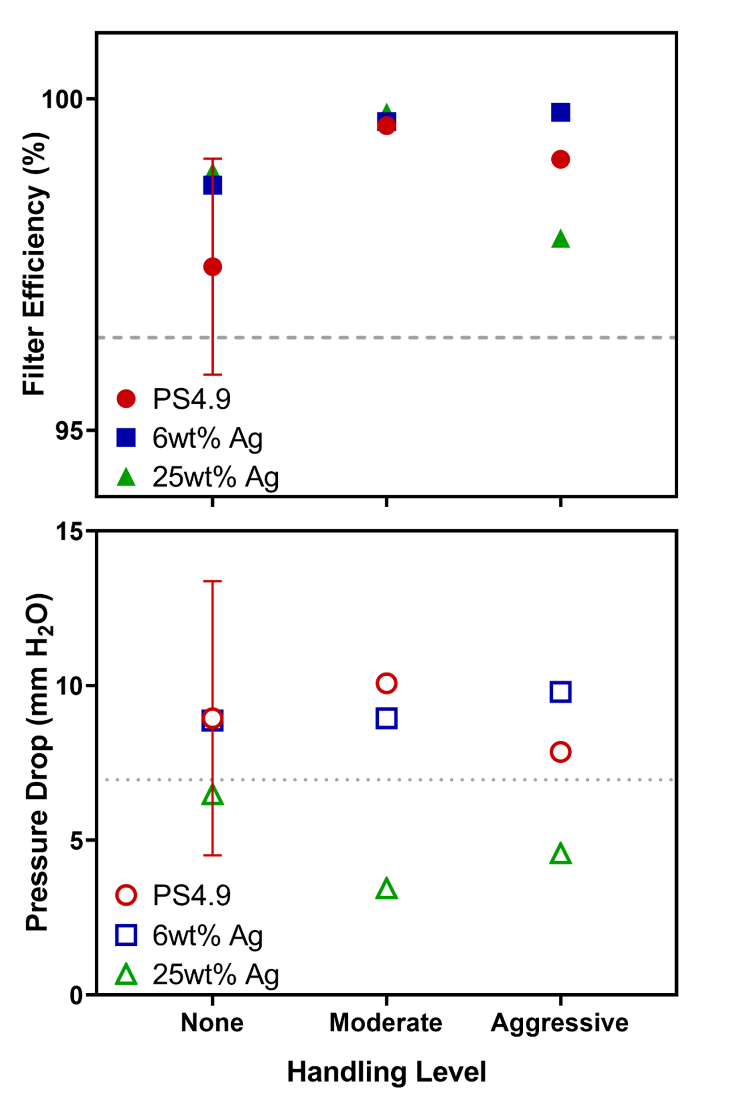


/PS

/PS

/PS

/PS

**A)**

**B)**

**Figure S9.** **(A)** Filtration efficiency and **(B)** pressure drop of PS_4.9_, 6 wt% Ag/PS, and 25wt% Ag/PS filter materials after moderate and aggressive handling. Also provided for comparison are the filter efficiency (96.4%) and pressure drop (6.96 mmH_2_O) measured for an N95 FFR (dashed line in each panel), as well as results obtained with pristine (unhandled) filter materials. All three filter materials exhibit higher filtration efficiency after moderate handling, likely due to material compaction, while the pressure drop across the materials after moderate handling remain comparable or lower (in the case of the 25 wt% Ag/PS filter) to that measured for pristine materials. Similar results are seen for the PS_4.9_ and 6 wt% Ag/PS filters after aggressive handling and deterioration. The 25 wt% Ag/PS filter material exhibits a lower pressure drop after aggressive handling, while filtration efficiency is slightly decreased but still well above 95% after aggressive handling.

**Table S5**. Surface area coverage of AgNP (AgNP%) for 25 wt% Ag-amended PS filters following handling procedures.

| Material | Handling Level | Avg. AgNP% |
| --- | --- | --- |
| 25 wt% Ag/PS | Pristine (no handling) | 8.6 ± 0.9 |
|  | Moderate | 6.2 ± 1.9 |
|  | Aggressive | 9.1 ± 1.2 |

**
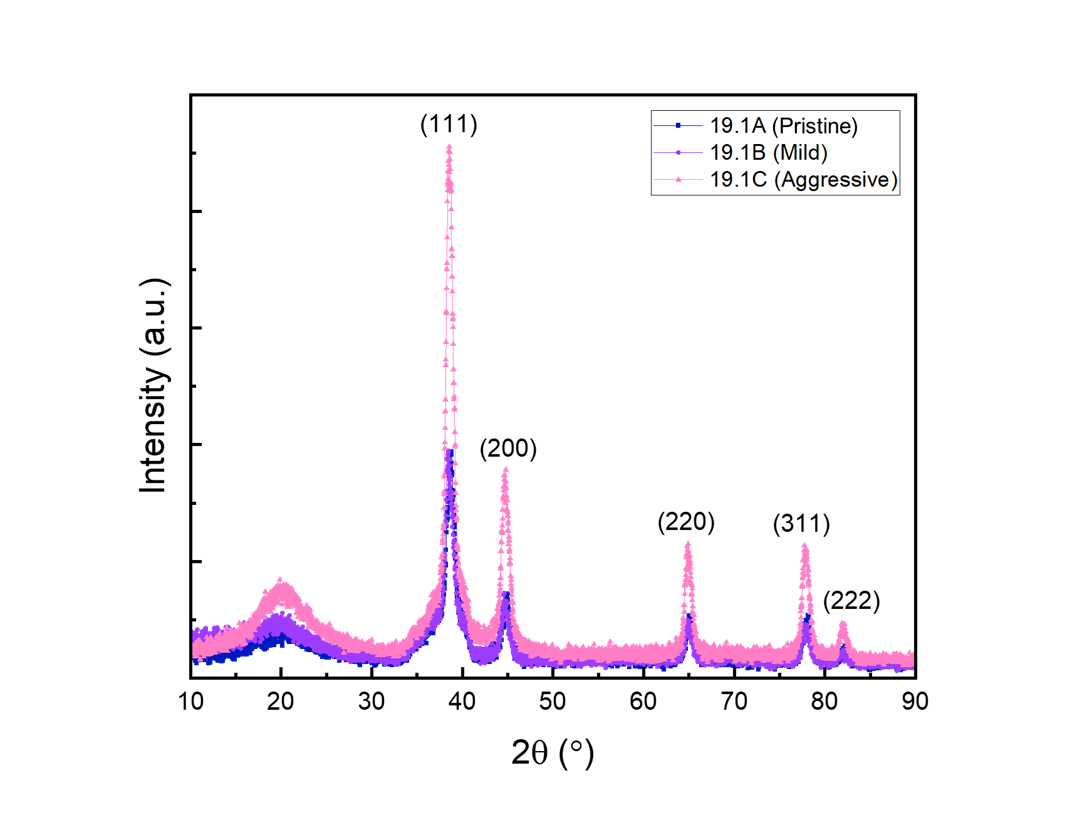
**

**Figure S10.** XRD patterns of 25 wt% Ag/PS filter material in pristine condition (no handling) and after moderate and aggressive handling procedures. The peaks located at the 2θ values of 38.49°, 44.73°, 64.91°, 77.88°, 82.07° are labeled with the corresponding Miller indices, which match the cubic crystalline planes of metallic Ag (Zhang et al. 2013). The XRD patterns demonstrate the strong presence of AgNPs in all three handled filters, consistent with no significant loss of AgNPs from the handling procedures (as suggested from complementary SEM analysis; see **Table S-4**).

**References**

Akduman, C. 2019. “Cellulose Acetate and Polyvinylidene Fluoride Nanofiber Mats for N95 Respirators.” *Journal of Industrial Textiles* 50 (8): 1239–61. https://doi.org/10.1177/1528083719858760.

Bałazy, A., M.Toivola, T. Reponen, A. Podgo´rski, P.Podgo´rski, A. Zimmer, and S. A. Grinshpun. 2006. "Manikin-based performance evaluation of N95 filtering-facepiece respirators challenged with nanoparticles." *The Annals of Occupational Hygiene* 50 (3): 259–269. doi: 10.1093/ANNHYG/MEI058.

Brown, R. C. 1993. *Air filtration: An integrated approach to the theory and applications of fibrous filters*. Oxford: Pergamon Press.

Chen, C., M. Lehtimäki, and K. Willeke. 1993. "Loading and filtration characteristics of filtering facepieces." *American Industrial Hygiene Association Journal* 54 (2): 51–60. doi: 10.1080/15298669391354324.

Choi, Sejin, Hyeonyeol Jeon, Min Jang, Hyeri Kim, Giyoung Shin, Jun Mo Koo, Minkyung Lee, et al. 2021. “Biodegradable, Efficient, and Breathable Multi-Use Face Mask Filter.” *Advanced Science* 8 (6): 2003155. https://doi.org/10.1002/ADVS.202003155.

Cramer, A. K., D. Plana, H. Yang, M. M. Carmack, E. Tian, M. S. Sinha, D. Krikorian, et al. 2021. "Analysis of SteraMist ionized hydrogen peroxide technology in the sterilization of N95 respirators and other PPE." *Scientific Reports* 11 (1): 1–10. doi: 10.1038/s41598-021-81365-7.

Davies, C.N. 1953. The separation of airborne dust and particles. *Proceedings of the Institution of Mechanical Engineers* 167 (1b): 185–213.

Deng, Chao, Farzad Seidi, Qiang Yong, Xiangyu Jin, Chengcheng Li, Xing Zhang, Jingquan Han, et al. 2022. “Antiviral/Antibacterial Biodegradable Cellulose Nonwovens as Environmentally Friendly and Bioprotective Materials with Potential to Minimize Microplastic Pollution.” *Journal of Hazardous Materials* 424 (February): 127391. https://doi.org/10.1016/j.jhazmat.2021.127391.

Greenstein, K. E., N. V. Myung, G. F. Parkin, and D. M. Cwiertny. 2019. "Performance comparison of hematite (Α-Fe2O3)-polymer composite and core-shell nanofibers as point-of-use filtration platforms for metal sequestration." *Water Research* 148: 492–503. doi: 10.1016/j.watres.2018.10.048.

He, Peiwen, Fan Wu, Ming Yang, Wenling Jiao, Xia Yin, Yang Si, Jianyong Yu, and Bin Ding. 2021. “Green and Antimicrobial 5-Bromosalicylic Acid/Polyvinyl Butyral Nanofibrous Membranes Enable Interception-Sterilization-Integrated Bioprotection.” *Composites Communications* 25 (6): 100720. https://doi.org/10.1016/j.coco.2021.100720.

Hinds, W. C. 1999. *Aerosol Technology: Properties, Behavior, and Measurement of Airborne Particles*. 2nd ed. New York: John Wiley and Sons.

Huang, Chengbo, Ying Liu, Zhiguang Li, Rong Li, Xuehong Ren, and Tung-Shi Huang. 2019. “N-Halamine Antibacterial Nanofibrous Mats Based on Polyacrylonitrile and N-Halamine for Protective Face Masks.” *Journal of Engineered Fibers and Fabrics* 14 (May): 1–8. https://doi.org/10.1177/1558925019843222.

Huang, L., S. Xu, Z. Wang, K. Xue, J. Su, Y.Song, S. Chen, C. Zhu, B. Z. Tang, and R. Ye. 2020. "Self-reporting and photothermally enhanced rapid bacterial killing on a laser-induced graphene mask." *ACS Nano* 14 (9): 12045-12053. doi: 10.1021/ACSNANO.0C05330.

Jung, Seojin, Jaejin An, Hyungjin Na, and Jooyoun Kim. 2019. “Surface Energy of Filtration Media Influencing the Filtration Performance against Solid Particles, Oily Aerosol, and Bacterial Aerosol.” *Polymers* 11 (6): 935. https://doi.org/10.3390/POLYM11060935.

Kang, Le, Yuankun Liu, Liping Wang, and Xiaoping Gao. 2021. “Preparation of Electrospun Nanofiber Membrane for Air Filtration and Process Optimization Based on BP Neural Network.” *Materials Research Express* 8 (11): 115010. https://doi.org/10.1088/2053-1591/AC37D6.

Kao, Tzu-Hao, Shuenn-Kung Su, Ching-Iuan Su, Ai-Wei Lee, and Jem-Kun Chen. 2016. “Polyacrylonitrile Microscaffolds Assembled from Mesh Structures of Aligned Electrospun Nanofibers as High-Efficiency Particulate Air Filters.” *Aerosol Science and Technology* 50 (6): 615–25. https://doi.org/10.1080/02786826.2016.1171822.

Kim, Juyoung, Seung Chan Hong, Gwi Nam Bae, and Jae Hee Jung. 2017. “Electrospun Magnetic Nanoparticle-Decorated Nanofiber Filter and Its Applications to High-Efficiency Air Filtration.” *Environmental Science and Technology* 51 (20): 11967–75. https://doi.org/10.1021/ACS.EST.7B02884.

Leung, Wallace Woon Fong, and Qiangqiang Sun. 2020. “Electrostatic Charged Nanofiber Filter for Filtering Airborne Novel Coronavirus (COVID-19) and Nano-Aerosols.” *Separation and Purification Technology* 250 (November): 116886. https://doi.org/10.1016/J.SEPPUR.2020.116886.

Li, Yuyao, Xia Yin, Yang Si, Jianyong Yu, and Bin Ding. 2020. “All-Polymer Hybrid Electret Fibers for High-Efficiency and Low-Resistance Filter Media.” *Chemical Engineering Journal* 398: 125626. https://doi.org/10.1016/j.cej.2020.125626.

Maze, B., H. V. Tafreshi, Q. Wang, and B. Pourdeyhimi. 2007. "A simulation of unsteady-state filtration via nanofiber media at reduced operating pressures." *Journal of Aerosol Science* 38 (5): 550–571. doi: 10.1016/J.JAEROSCI.2007.03.008.

NIOSH (National Institute for Occupational Safety and Health). 2019. Determination of particulate filter efficiency level for N95 series filters against solid particulates for non-powered, air-purifying respirators Standard Testing Procedure (STP). Procedure No. TEB-APR-STP-0059. Revision 3.2, National Personal Protective Technology Laboratory, Pittsburgh, PA. Accessed September 07, 2021. https://www.cdc.gov/niosh/npptl/stps/pdfs/TEB-APR-STP-0059-508.pdf

O’Shaughnessy, P.T., B. Strzelecki, M. Ortiz-Hernandez, P. Aubin, X. Jing, Q. Chang, J. Xiang, P. S. Thorne, and J.T. Stapleton. 2021. "Characterization of performance and disinfection resilience of nonwoven filter materials for use in 3D-printed N95 respirators." *Journal of Occupational and Environmental Hygiene* 18 (6): 265–275. doi: 10.1080/15459624.2021.1913283.

Pais, Vânia, Carlos Mota, João Bessa, José Guilherme Dias, Fernando Cunha, and Raul Fangueiro. 2021. “Study of the Filtration Performance of Multilayer and Multiscale Fibrous Structures.” *Materials 2021* 14 (23): 7147. https://doi.org/10.3390/MA14237147.

Peter, K. T., A. J. Johns, N. V. Myung, and D. M. Cwiertny. 2017. "Functionalized polymer-iron oxide hybrid nanofibers: Electrospun filtration devices for metal oxyanion removal." *Water Research* 117: 207–17. doi: 10.1016/j.watres.2017.04.007.

Peter, K. T., J.D. Vargo, T. P. Rupasinghe, A. De Jesus, A. V. Tivanski, E. A. Sander, N. V. Myung, and D. M. Cwiertny. 2016. "Synthesis, optimization, and performance demonstration of electrospun carbon nanofiber–carbon nanotube composite sorbents for point-of-use water treatment." *ACS Applied Materials & Interfaces* 8 (18): 11431-11440. doi: 10.1021/acsami.6b01253.

Qian, J., B. Jennings, D. M. Cwiertny, and A. Martinez. 2017. "Emerging investigator series: Development and application of polymeric electrospun nanofiber mats as equilibrium-passive sampler media for organic compounds." *Environmental Science: Processes and Impacts* 19 (11): 1445-1456. doi: 10.1039/c7em00289k.

Reyes, Catherine G., and Margaret W. Frey. 2017. “Morphological Traits Essential to Electrospun and Grafted Nylon-6 Nanofiber Membranes for Capturing Submicron Simulated Exhaled Breath Aerosols.” *Journal of Applied Polymer Science* 134 (17): 44759. https://doi.org/10.1002/APP.44759.

Ruan, Dongliang, Liming Qin, Rouxi Chen, Guojie Xu, Zhibo Su, Jianhua Cheng, Shilei Xie, Faliang Cheng, and Frank Ko. 2020. “Transparent PAN:TiO2 and PAN-Co-PMA:TiO2 Nanofiber Composite Membranes with High Efficiency in Particulate Matter Pollutants Filtration.” *Nanoscale Research Letters* 15 (1): 1–8. https://doi.org/10.1186/S11671-019-3225-2.

Saikaew, Rateeya, and Varol Intasanta. 2021. “Versatile Nanofibrous Filters against Fine Particulates and Bioaerosols Containing Tuberculosis and Virus: Multifunctions and Scalable Processing.” *Separation and Purification Technology* 275 (November): 119171. https://doi.org/10.1016/J.SEPPUR.2021.119171.

Swamidoss, V. Felix, Mohan Bangaru, Gobi Nalathambi, Dharmalingam Sangeetha, and Arun Karthick Selvam. 2018. “Silver-Incorporated Poly Vinylidene Fluoride Nanofibers for Bacterial Filtration.” *Aerosol Science and Technology* 53 (2): 196–206. https://doi.org/10.1080/02786826.2018.1554892.

TOMI Environmental Solutions, Inc. n.d. The IHP^TM^ Process. Accessed January 22, 2021. https://tomimist.com/about/ihp/.

USEPA Office of Science. 2001. *Method 1602: Male-Specific (F + ) and Somatic Coliphage in Water by Single Agar Layer (SAL) Procedure*. EPA Office of Water, Washington, D.C. Accessed August 02, 2021. https://www.epa.gov/sites/default/files/2015-12/documents/method_1602_2001.pdf.

Vanangamudi, Anbharasi, Sakinah Hamzah, and Gurdev Singh. 2015. “Synthesis of Hybrid Hydrophobic Composite Air Filtration Membranes for Antibacterial Activity and Chemical Detoxification with High Particulate Filtration Efficiency (PFE).” *Chemical Engineering Journal* 260 (January): 801–8. https://doi.org/10.1016/J.CEJ.2014.08.062.

Wang, Chenrong, Wei Wang, Lishan Zhang, Shan Zhong, and Dan Yu. 2019. “Electrospinning of PAN/Ag NPs Nanofiber Membrane with Antibacterial Properties.” *Journal of Materials Research* 34 (10): 1669–77. https://doi.org/10.1557/JMR.2019.44.

Welch, J. L., J. Xiang, S. R. Mackin, S. Perlman, P. Thorne, P. O’Shaughnessy, B. Strzelecki, P. Aubin, M. Ortiz-Hernandez, and J. T. Stapleton. 2021. "Inactivation of severe acute respiratory coronavirus virus 2 (SARS-CoV-2) and diverse RNA and DNA viruses on three-dimensionally printed surgical mask materials." *Infection Control & Hospital Epidemiology* 42 (3): 253–260. doi: 10.1017/ICE.2020.417.

Zhang, N., X. Yu, J. Hu, F. Xue, and E. Ding. 2013. "Synthesis of silver nanoparticle-coated poly(styrene-co-sulfonic acid) hybrid materials and their application in Surface-Enhanced Raman Scattering (SERS) tags.” *RSC Advances* 3 (33): 13740-13747. doi: 10.1039/C3RA40888D.

Zhu, Miaomiao, Dawei Hua, Ming Zhong, Lingfeng Zhang, Fang Wang, Buhong Gao, Ranhua Xiong, and Chaobo Huang. 2018. “Antibacterial and Effective Air Filtration Membranes by ‘Green’ Electrospinning and Citric Acid Crosslinking.” *Colloid and Interface Science Communications* 23 (March): 52–58. https://doi.org/10.1016/J.COLCOM.2018.01.002.

Zhang, Shichao, Hui Liu, Xia Yin, Jianyong Yu, and Bin Ding. 2016. “Anti-Deformed Polyacrylonitrile/Polysulfone Composite Membrane with Binary Structures for Effective Air Filtration.” *ACS Applied Materials and Interfaces* 8 (12): 8086–95. https://doi.org/10.1021/ACSAMI.6B00359.

Zhang, Shichao, Ning Tang, Leitao Cao, Xia Yin, Jianyong Yu, and Bin Ding. 2016. “Highly Integrated Polysulfone/Polyacrylonitrile/Polyamide-6 Air Filter for Multilevel Physical Sieving Airborne Particles.” *ACS Applied Materials and Interfaces* 8 (42): 29062–72. https://doi.org/10.1021/ACSAMI.6B10094.

Zhu, Zan, Yu Zhang, Liang Bao, Jianping Chen, Shun Duan, Sheng-Chieh Chen, Ping Xu, and Wei-Ning Wang. 2021. “Self-Decontaminating Nanofibrous Filters for Efficient Particulate Matter Removal and Airborne Bacteria Inactivation.” *Environmental Science: Nano* 8 (4): 1081–95. https://doi.org/10.1039/D0EN01230K.

Zolotarev, V.M. 2017. Comparison of polystyrene IR spectra obtained by the T, R, ATR, and DR methods. *Condensed-Matter Spectroscopy* 122, 749-756.

1. Present affiliation for YYC: Department of Chemical and Biomolecular Engineering, University of Notre Dame, Notre Dame, IN 46556, United States [↑](#footnote-ref-2)
